# Species-Discriminating Diagnostic PCR, Ribosomal Intergenic Spacer-Based Single-Marker Taxonomy and Cryptic Descriptions of the Fungal Entomopathogens *Metarhizium hybridum* and *Metarhizium parapingshaense*

**DOI:** 10.3390/jof12040272

**Published:** 2026-04-09

**Authors:** Christina Schuster, Haifa Ben Gharsa, Yamilé Baró Robaina, Romina G. Manfrino, Saikal Bobushova, Alejandra C. Gutierrez, Claudia C. López Lastra, Andreas Leclerque

**Affiliations:** 1 Department of Biology, Technische Universität Darmstadt (TUDa), Schnittspahnstraße 10, 64287 Darmstadt, Germanyhaifabengharsa@gmail.com (H.B.G.); 2 Plant Health Research Institute (INISAV), 110 Str. 514, Havana 11600, Cuba; robainabaro@gmail.com; 3 Centro de Estudios Parasitológicos y de Vectores (CEPAVE), CONICET-Consejo Nacional de Investigaciones Científicas y Técnicas, UNLP-Universidad Nacional de La Plata, La Plata 1900, Buenos Aires, Argentina; gutierrez@cepave.edu.ar (A.C.G.); claudia@cepave.edu.ar (C.C.L.L.); 4 Instituto de Investigación de la Cadena Láctea (IDICAL), CONICET-Consejo Nacional de Investigaciones Científicas y Técnicas, INTA-Instituto Nacional de Tecnología Agropecuaria, Rafaela 2300, Santa Fe, Argentina; manfrino.romina@inta.gob.ar; 5 Faculty of Agriculture, Kyrgyz-Turkish Manas University, 56 Chyngyz Aitmatov Avenue, Bishkek 720038, Kyrgyzstan; saykal.bobusheva@manas.edu.kg

**Keywords:** *Metarhizium hybridum*, *Metarhizium parapingshaense*, *Metarhizium anisopliae sensu lato*, *Metarhizium pingshaense sensu lato*, PARB clade, ribosomal intergenic spacer marker (rIGS-ID800), single-marker phylogeny, sequencing-independent species identification, diagnostic PCR, cryptic species descriptions

## Abstract

(1) Background: Potentially arthropod-pathogenic and plant-associated *Metarhizium* fungi are of high interest for basic research, biological pest control and plant growth promotion. Unambiguous species delineation enabling the taxonomic assignment of new isolates and the identification of new *Metarhizium* species is of crucial importance for both research and application. Recently, the new species *Metarhizium hybridum* and *Metarhizium parapingshaense* were introduced on the basis of phylogenomic studies. (2) Methods: Neighbor- joining and Bayesian inference-based phylogenetic reconstruction of ribosomal intergenic spacer (rIGS) sequences were used to critically evaluate new species introductions. A species-discriminating diagnostic PCR tool for *Metarhizium* was adapted to *M. hybridum* and *M. parapingshaense*. GenBank database mining was performed to identify cryptic descriptions of the new species. (3) Results: The introduction of *M. hybridum* and *M. parapingshaense* was corroborated by rIGS sequence comparison. Data mining revealed cryptic first descriptions of *M. hybridum* from Canada, China, Colombia, Costa Rica, Cuba, Honduras, Mexico, New Zealand, the USA and the Philippines, and of *M. parapingshaense* from China, India, Japan, the Philippines and South Korea. (4) Conclusions: Results support the reliability of rIGS as a single taxonomic marker for species-level identification of *Metarhizium* fungi. Species-discriminating diagnostic PCR was successfully adapted to enable the sequencing-independent identification of the confirmed new species *M. hybridum* and *M. parapingshaense*.

## 1. Introduction

The taxonomic genus *Metarhizium* Sorokin (Hypocreales; Clavicipitaceae) comprises natural fungal pathogens of numerous economically relevant insect hosts and is among the fungi most widely investigated for and applied in biological control. The biocontrol potential of *Metarhizium* fungi has been successfully exploited to develop microbial insecticides [1,2]. In addition to its role as an entomopathogen, multiple studies have documented beneficial plant–fungus interactions in some *Metarhizium* species, including rhizosphere and endophytic colonization that can enhance plant nutrient acquisition and growth, improve resistance to biotic and abiotic stressors, and facilitate the mobilization of soil nutrients such as phosphorus and trace metals, thereby functioning as a plant growth promoter and bioinoculant in agroecosystems [3]. Review and empirical studies also report increased biomass, root elongation, and physiological performance in several crops following *Metarhizium* inoculation, supporting its dual use as both a biocontrol agent and a plant growth-promoting symbiont [4]. Elucidation of lineage diversification has revealed that broad-host-range clades derived from plant-associated ancestors exhibit marked ecological plasticity, enabling both insect pathogenicity and plant associations [5]. Consequently, precise taxonomic resolution is fundamental not only for regulatory and applied biocontrol purposes, but also for the strategic selection and deployment of isolates as multifunctional agents with plant growth-promoting potential.

Traditionally, species assignment within the genus *Metarhizium* has been based on morphological and morphometric traits such as colony morphologies and the dimensions of conidia, conidiophores and phialides. During the past decades, these morphological criteria have been complemented by the use of genetic markers such as the Internal Transcribed Spacer (ITS) sequence of the fungal rRNA operon [6], a Multilocus Sequence Analysis (MLSA) scheme comprising the genes encoding translation elongation factor 1 alpha (EF1A) and the RNA polymerase II subunits 1 and 2 (RPB1 and RPB2, respectively) and the intron-rich 5′-region of the translation elongation factor 1 alpha gene, referred to as “5TEF” [7,8]. Moreover, a nuclear intergenic region termed MzIGS3 or DUF895 [9] has been successfully employed in both species delineation and diversity studies [4,10,11], and the intergenic spacer sequence of the ribosomal RNA operon (rIGS) has been evaluated as a molecular taxonomic marker for *Metarhizium* fungi [12,13,14].

Molecular taxonomy has led to the discovery of cryptic or hidden species, i.e., monophyletic groups of organisms that are morphologically or phenotypically indistinguishable, but genetically diverse from a known species [15]. In the case of *Metarhizium* fungi that are characterized by only subtle morphological traits, systematic studies employing genetic markers led to the redefinition of genus boundaries and species delineations [16,17,18] and to the introduction of new *Metarhizium* species [19,20,21,22,23]. In particular, the reorganized genus *Metarhizium* was found to comprise a tight cluster, informally termed the “PARB clade” [18], of the five species *Metarhizium anisopliae, M. brunneum*, *M. humberi, M. pingshaense* and *M. robertsii* that are of high relevance for both fundamental research and insect biocontrol.

Recently, a rIGS sequence-based species-discriminating diagnostic PCR protocol for the sequencing-independent species-level identification has been established [13] and successfully applied to introduce the new PARB clade species, *Metarhizium caribense* [24]. Contemporaneously, an integrative taxonomic study by Kobmoo et al. (2024) combining phylogenomics with phenotypic data from morphometrics, metabolomics and insect virulence bioassays, led to the delineation of two further PARB clade species, *Metarhizium hybridum* and *Metarhizium parapingshaense,* from *M. anisopliae* and *M. pingshaense*, respectively [25]. The study described three *M. parapingshaense* strains from Thailand and the Solomon Islands, as well as a single *M. hybridum* strain from Brazil; subsequently, *M. hybridum* was reported from Argentina, too [26].

It was the purpose of the present study to validate the functionality of the rIGS sequence as a single taxonomic marker for species delineation within the reorganized PARB clade, to extend the species-discriminating PCR diagnostics to the new taxa *M. hybridum* and *M. parapingshaense*, and to identify possible cryptic descriptions of these new species.

Throughout this publication, “*M. anisopliae sensu stricto*” will be referred to in the usual way, i.e., as the taxonomic species defined by its nomenclatural type strain ARSEF 7487. A recent proposal to rename this taxon “*Metarhizium neoanisopliae*” [25] will not be adopted here as broader community acceptance is awaited. Moreover, the designation “*Metarhizium anisopliae sensu lato*” will be used to refer to a sub-structure of the PARB clade comprising the species *M. anisopliae sensu stricto* together with *M. humberi* and *M. hybridum.* Analogously, “*M. pingshaense sensu lato”* will designate a PARB sub-clade made up of the three species *M. pingshaense sensu stricto, M. caribense* and *M. parapingshaense.* If not otherwise stated, the species designations “*M. anisopliae*” and *“M. pingshaense*” will always refer to *M. anisopliae sensu stricto* and *M. pingshaense sensu stricto*, respectively.

## 2. Materials and Methods

### 2.1. DNA Extraction, PCR Amplification and DNA Sequence Determination

For DNA extraction, *Metarhizium* fungi investigated in this study (Supplementary Table S1) were grown for up to 5 days at 24 °C in 10 mL YPG liquid medium (2 g/L yeast extract, 10 g/L peptone, 20 g/L glucose) containing 25 µg/mL of tetracycline. Approximately 200–300 mg of fungal biomass were transferred to a 2 mL screw-capped microcentrifuge tube containing Lysing Matrix C (MP Biomedicals, Irvine, CA, USA). Samples were frozen at −20 °C for at least 3 h and processed for 60 s at intermediate speed in a Minilys homogenizer (Bertin technologies, Montigny-le-Bretonneux, France). DNA was extracted from homogenized samples using the DNeasy Plant Mini kit (Qiagen, Venlo, The Netherlands) according to the standard protocol provided by the manufacturer, starting with the addition of 400 µL AP1 buffer. DNA was eluted from the column in 100 µL AE buffer (10 mM Tris-Cl, 0.5 mM EDTA, pH = 9.0) and stored at −20 °C. DNA concentrations were determined using a NanoDrop One spectrophotometer (Thermo Scientific, Waltham, MA, USA).

PCR amplifications were run in a T-One thermocycler (Biometra, Göttingen, Germany) using 0.025 U/µL GoTaq polymerase (Promega, Fitchburg, MA, USA) with dNTP and oligonucleotide primer concentrations of 200 µM and 500 nM, respectively.

Preparative PCR amplifications for DNA sequence determination were performed as 50 µL reactions using a generalized PCR protocol. An initial denaturation step of 95 °C for 2 min was followed by 35 cycles consisting of a 30 s denaturation step at 95 °C, a 30 s annealing step and an elongation step at 72 °C. Primer pair-specific annealing temperatures and amplicon-specific elongation times were indicated in the Supplementary Table S2. The program ended with a 5 min final elongation step at 72 °C. Using this protocol, both the complete rIGS sequence and the rIGS-ID800 marker were amplified using primer pairs Ma-28S4f/Ma-18S4r and Migs1-F1/Migs850-R1, respectively (Supplementary Table S2). Formation of single PCR products of expected apparent size was routinely controlled through horizontal electrophoresis in 1x TAE buffer (40 mM TRIS, 20 mM acetic acid, 1 mM EDTA, pH 8.3) of a 5 µL sample using 1% agarose gels stained with 5 µL/100 mL Roti GelStain (Carl Roth, Karlsruhe, Germany). PCR products were purified using the Qiaquick PCR purification kit (Qiagen, Venlo, The Netherlands) according to the standard protocol provided by the manufacturer. Purified PCR products were eluted for direct sequencing in 50 µL of EB buffer (10 mM Tris·Cl, pH 8.5) and stored at −20 °C.

Sanger sequencing of rIGS-ID800 PCR products using primers Migs1-F1 and Migs850-R1, as well as Oxford Nanopore Technology sequencing of complete amplified rIGS regions, was performed by an external provider (MicroSynth-SeqLab, Gießen, Germany). rIGS-ID800 raw sequence data were combined into a consensus sequence for each fungal specimen using version 11 of the MEGA software package [27]. Sequences determined were submitted to the GenBank database under the accession numbers indicated in the Supplementary Table S1.

Diagnostic PCR amplifications for sequencing-independent species discrimination were performed as 20 µL reactions containing a working concentration of 100 pg/µL of DNA template. The diagnostic PCR program consisted of one initial denaturation step of 95 °C for 2 min, 35 cycles of denaturation at 95 °C for 30 s, annealing at 60 °C for 30 s, and elongation at 72 °C for 45 s, followed by a 2 min final elongation step at 72 °C [13].

### 2.2. Data Mining

Whole-length rIGS sequences of *M. parapingshaense* strains BCC 37941 and BCC 96582 were assembled from published SRA data (accession number PRJNA1111679), running a targeted BlastN search for reads mapping the complete rIGS sequence of *M. parapingshaense* reference strain ARSEF 4342. Identified reads were assembled using the Clustal X function of the MEGA 11 software package. Potential insertions or deletions with respect to the query sequence were corroborated by SRA data searches with joint flanking sequences.

A total of 46 assembled genome sequences assigned to the genus *Metarhizium* in the GenBank database (https://www.ncbi.nlm.nih.gov/datasets/genome/?taxon=5529, accessed on 3 February 2026) were searched by the genomic Blast option for cryptic descriptions of *M. hybridum* or *M. parapingshaense* using as query complete rIGS sequences of the specific type strains ARSEF 549 and BCC 37941. Results were cross-checked by analogous searches with 5TEF marker sequences of ARSEF 549 and the *M. parapingshaense* reference strain ARSEF 4342 (Supplementary Table S1).

Homologous single-marker entries to the GenBank database were searched for using the BlastN software tool (https://blast.ncbi.nlm.nih.gov/Blast.cgi?PROGRAM=blastn&PAGE_TYPE=BlastSearch&LINK_LOC=blasthome, accessed on 2 February 2026) [28,29]. Searches for cryptic descriptions of the new species *M. hybridum* or *M. parapingshaense* were performed using the rIGS-ID800 sequences of the type strains ARSEF 549 and BCC 37941 as query. In a second step, results were complemented by BlastN searches using the DUF895, 5TEF, EF1A and RPB2 marker sequences of strains *M. hybridum* ARSEF 549 and *M. parapingshaense* ARSEF 4342 as query (Supplementary Table S1). GenBank entries displaying a minimum of 90% sequence coverage with respect to the corresponding query sequence were retained for downstream analyses.

GenBank entries retained from both genomic and single-entry searches were aligned with respective reference sequences to reconstruct NJ phylogenies. Fungal strains or isolates clustering in the tree topology with the *M. hybridum* or the *M. parapingshaense* reference strains under unambiguous delineation from the further species of, respectively, *M. anisopliae sensu lato* and *M. pingshaense sensu lato*, were considered cryptic descriptions of the two species.

### 2.3. Phylogenetic Reconstruction

Nucleotide sequence alignments were generated using the CLUSTAL W function [30] as implemented in MEGA 11. Then, p-distance matrix-based neighbor-joining (NJ) phylogenies were reconstructed under pairwise deletion of alignment gaps and missing data. Tree topology confidence limits were explored in non-parametric bootstrap analyses over 1000 pseudo-replicates. Moreover, Bayesian phylogenetic hypotheses were explored using version 3.2.7 of the MrBayes software tool [31] under a GTR + I model assuming a gamma-shaped distribution of rates across sites and allowing for eight rate categories. Prior probabilities were estimated from the data set. Two runs comprising each one cold and three heated MCMC chains were performed in parallel over 1,000,000 generations, sampling every 5000 generations. A standard deviation of split frequencies below 0.01 was applied as a convergence criterion. Trees from the first 25% of generations were discarded as burn-in prior to the generation of a 50% majority rule consensus tree.

## 3. Results

### 3.1. rIGS-Based Phylogeny for M. hybridum and M. parapingshaense

Complete rIGS sequences were determined for those *M. hybridum, M. anisopliae, M. parapingshaense* and *M. pingshaense* strains that were compared at the whole genome level by Kobmoo et al. (2024) [25] when introducing *M. hybridum* and *M. parapingshaense*. In particular, complete rIGS sequences were assembled from WGS sequence read data deposited in the GenBank database for strains *M. parapingshaense* BCC 37941-T and BCC 96582, *M. hybridum* ARSEF 549-T, and *M. anisopliae* ARSEF 2080, giving rise to continuous rIGS sequences comprising 1555 bp, 1543 bp, 1708 bp and 1750 bp, respectively. Moreover, for strains ARSEF 549 and ARSEF 2080, rIGS sequence assemblies were confirmed by PCR amplification and de novo sequencing of the rIGS region. GenBank accession numbers of complete rIGS sequences were indicated in the Supplementary Table S1.

**Figure 1:**
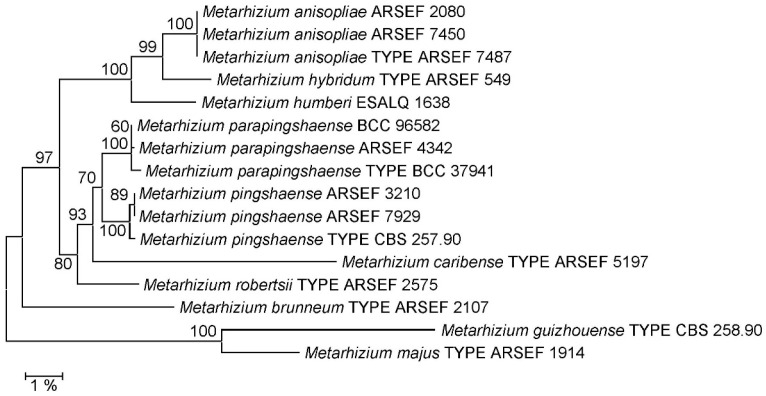
Neighbor-joining (NJ) phylogeny of *Metarhizium* fungi as reconstructed from complete ribosomal intergenic spacer (rIGS) sequences. Terminal branches are labeled by genus, species and strain designations; “TYPE” denotes the nomenclatural type strain of a species. Numbers on branches indicate bootstrap support percentages. The size bar corresponds to 1% sequence divergence. The orthologous sequences from the *M. majus* and *M. guizhouense* type strains were used as outgroup.

Comparison to the complete rIGS sequences of nomenclatural type and reference strains representing all *Metarhizium* species currently described in the PARB clade (Supplementary Table S1) gave rise to trees of identical topology irrespective of the method—neighbor-joining (Figure 1) or Bayesian inference (Supplementary Figure S1)—used for phylogenetic reconstruction. With respect to species delineation within the PARB clade, this tree topology was identical to the topological structure of the PARB clade in the phylogeny generated by Kobmoo et al. (2024) [25] from a concatenated matrix of 237 genes, with the relevant difference that species *M. humberi* and *M. caribense* were not represented in this whole genome tree. Importantly, delineations of the new species *M. hybridum* from *M. anisopliae* and *M. parapingshaense* from *M. pingshaense* as proposed by Kobmoo et al. (2024) [25] received 100% bootstrap support or posterior probabilities of 1.0 in the rIGS-based NJ and BI phylogenies, respectively. Moreover, *M. hybridum* and *M. parapingshaense* were delineated from their respective second neighboring species, *M. humberi* and *M. caribense*, by 100% bootstrap support or 1.0 posterior probability.

### 3.2. Cryptic Descriptions of M. hybridum and M. parapingshaense

Analysis of the 46 assembled *Metarhizium* whole genome sequences published in the GenBank database revealed one further *M. hybridum* genome—namely that of strain E6 currently assigned to *M. anisopliae*—in addition to the type strain ARSEF 549 genome. No assembled *M. parapingshaense* genomes were identified.

**Figure 2:**
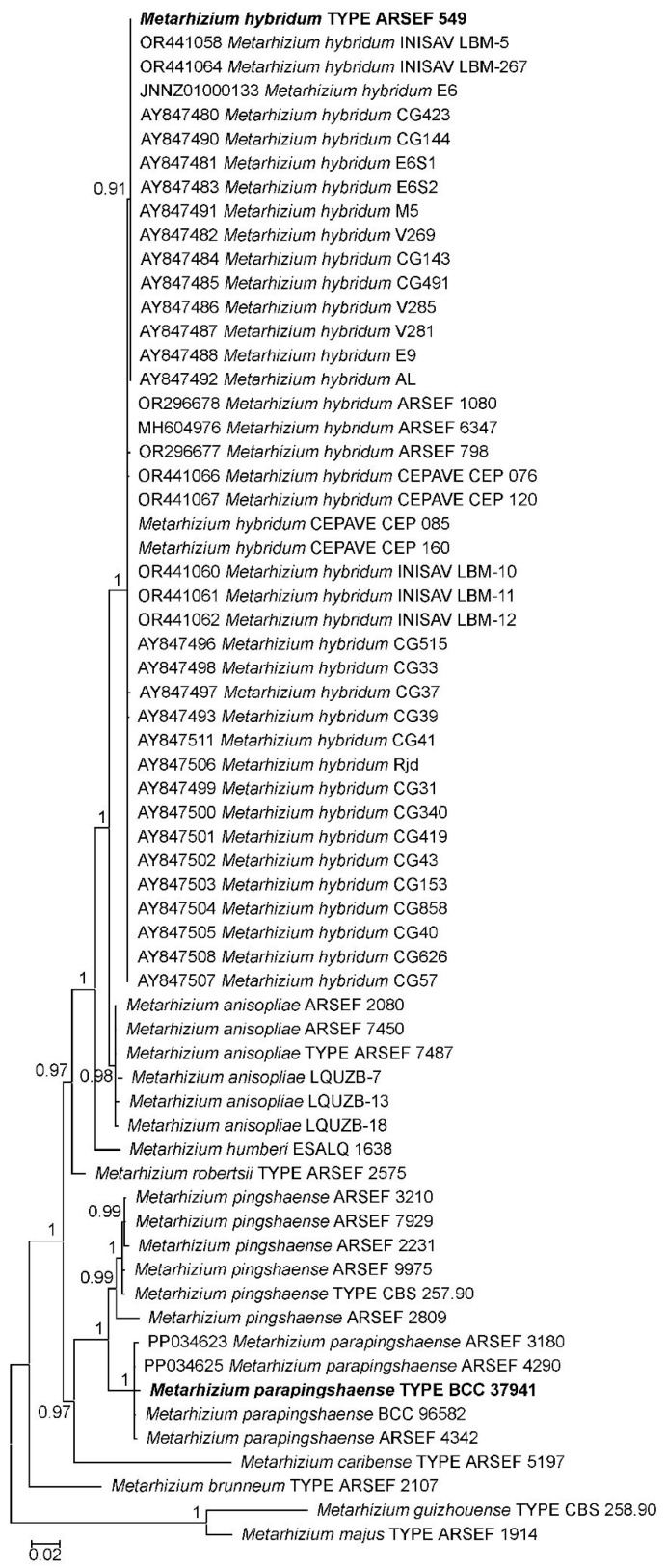
Bayesian inference (BI)-based phylogeny of *Metarhizium* fungi as reconstructed from rIGS-ID800 marker sequences. Terminal branches are labeled by genus, species and strain designations; “TYPE” denotes the nomenclatural type strain of a species. *M. hybridum* and *M. parapingshaense* type strains are displayed in bold face. GenBank accession numbers are indicated for cryptic descriptions identified in the GenBank database. Numbers on branches indicate posterior probability (pp) values. The size bar indicates the number of expected substitutions per site. The orthologous sequences from the *M. majus* and *M. guizhouense* type strains were used as outgroup.

Searches for rIGS-ID800 homologous single GenBank database entries revealed 37 cryptic descriptions of *M. hybridum* and two of *M. parapingshaense* (Figure 2, Supplementary Figure S2). Extension of the GenBank search to markers DUF895, 5TEF, EF1A, and RPB2 identified 77 further strains of *M. hybridum* and 28 of *M. parapingshaense* (Supplementary Table S3). Bootstrap support values for *M. hybridum* and *M. parapingshaense* clades varied strongly for the different marker–species combinations, being >95% with rIGSI-D800, DUF895 and RPB2, but only app. 65% with 5TEF and EF1A for *M. hybridum,* as well as >95% with 5TEF and DUF895, but only app. 85% with rIGS-ID800 and EF1A for *M. parapingshaense* (Supplementary Figures S2–S6).

Whereas most *M. hybridum* strains had previously been identified as *M. anisopliae* or *Metarhizium* sp., strains of *M. parapingshaense* had almost exclusively been assigned to *M. pingshaense* (Table 1). With respect to geographic origins, cryptic description data appeared severely biased towards the Americas for *M. hybridum* (104/115) and Asia/Oceania for *M. parapingshaense* (30/30). While *M. hybridum* was mainly isolated from associations with insects (77/115), soil appeared to be the most frequent isolation source for *M. parapingshaense* (23/30).

### 3.3. Species-Discriminating Diagnostic PCR for M. hybridum and M. parapingshaense

Building on the previous establishment of a species-discriminating PCR tool for the *Metarhizium* PARB clade [13], species-discriminating primer pairs for the delineation of *M. hybridum* and *M. anisopliae,* as well as *M. parapingshaense* and *M. pingshaense* were designed against alignments of complete rIGS sequences (Supplementary Figures S7 and S8) and optimized for functionality under pre-established PCR parameters. When the discriminative power of these primer pairs—termed mani-ID, mhyb-ID, mppi-ID and mpin-ID (Table 2) was tested in diagnostic PCR reactions with genomic DNA of reference strains representing the *Metarhizium* PARB clade species *M. anisopliae, M. brunneum, M. caribense, M. hybridum, M. parapingshaense, M. pingshaense* and *M. robertsii*, PCR products were uniquely generated from cognate DNA templates and were of expected apparent length (Supplementary Figure S9). Due to the unavailability of *M. humberi* DNA, discriminative power with respect to this species was not tested experimentally. However, in silico analyses comparing diagnostic primer pairs with the unique available *M. humberi* rIGS sequence from the ESALQ 1638 genome led to the prediction that no product would be amplified at least from this reference strain.

**Figure 3:**
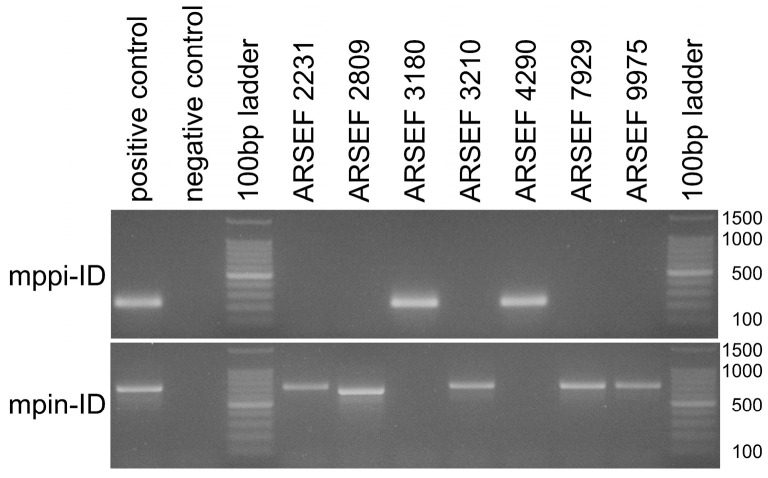
Agarose gel electrophoresis of diagnostic PCRs using species-discriminating primer pairs mpin-ID and mppi-ID specific for *M. pingshaense* and *M. parapingshaense*, respectively, as indicated at the left margin. The length (in bp) of the main signals in the size standard is indicated at the right margin. Lane labels on top of the gel pictures designate the *Metarhizium* strain or isolate; “positive control” denotes the primer pair-specific cognate type strain; “negative control” indicates the no template control; “100 bp ladder” denotes the size standard.

**Figure 4:**
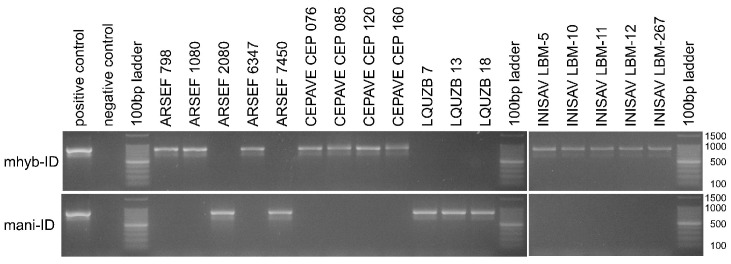
Agarose gel electrophoresis of diagnostic PCRs using species-discriminating primer pairs mani-I and mhyb-ID specific for *M. anisopliae and M. hybridum*, respectively, as indicated at the left margin. The length (in bp) of the main signals in the size standard is indicated at the right margin. Lane labels on top of the gel pictures designate the *Metarhizium* strain or isolate; “positive control” denotes the primer pair-specific cognate type strain; “negative control” indicates the no template control; “100 bp ladder” denotes the size standard.

The functionality and species-discriminative power of designed diagnostic primers were further validated with two sets of 17 and seven *Metarhizium* strains and isolates that were, respectively, supposed to be closely related to *M. anisopliae sensu lato* or *M. pingshaense sensu lato*. More exactly, a set of twelve strains of globally distributed geographic origins obtained from the ARSEF collection was complemented with four Argentine strains from the CEPAVE and five Cuban strains from the INISAV culture collections, as well as three isolates (designated LQUZB) stemming from Uzbekistan (Supplementary Table S1). When the extracted genomic DNA of these fungi was probed with the diagnostic primer pairs mani-ID and mhyb-ID for *M. anisopliae sensu lato* as well as mppi-ID and mpin-ID for *M. pingshaense sensu lato*, exactly one single reaction per template was found positive (Figure 3 and Figure 4), motivating the assignment of each strain to one of the respective four *Metarhizium* species. From a total of 24 fungal strains tested, five were thus found assignable to *M. anisopliae,* twelve to *M. hybridum,* two to *M. parapingshaense* and five to *M. pingshaense*. PCR amplification and determination of the rIGS-ID800 marker sequence fully corroborated these diagnostic PCR-based species-level assignments (Figure 2).

## 4. Discussion

As part of an integrative taxonomic study combining phylogenomics, morphometrics, metabolomics and virulence data, Kobmoo et al. (2024) had introduced two new taxa in the *Metarhizium* PARB clade [25]: phylogenetic reconstruction based on a concatenation of 237 selected fungal genes had delineated (i) the new species *M. hybridum* from *M. anisopliae sensu stricto* in *M. anisopliae sensu lato* and (ii) the new species *M. parapingshaense* from *M. pingshaense sensu stricto* in *M. pingshaense sensu lato.* In the present study, these species delineations were corroborated, receiving optimal support in both the NJ and BI phylogenies (Figure 1, Supplementary Figure S1) reconstructed from the complete ribosomal intergenic spacer sequences of the same fungal strains compared in the study cited.

This is a meaningful result as two further *Metarhizium* species that had previously been delineated within *M. anisopliae sensu lato* and *M. pingshaense sensu lato*, namely *M. humberi* [22] and *M. caribense* [24], were not considered in the description of *M. hybridum* and *M. parapingshaense*. In the present study, these taxa were represented in the analysis, and the above results confirm that (i) *M. hybridum* is a new species with respect not only to *M. anisopliae sensu stricto*, but also to *M. humberi,* and that (ii) *M. parapingshaense* is correctly delineated not only from *M. pingshaense sensu stricto,* but also from *M. caribense.*

In turn, the reproducibility of new species delineations that were introduced from an integrative geno- and phenotypic approach, in the rIGS-based phylogenetic reconstruction, lends strong support to the rIGS-based single-marker approach at this taxonomic level. It has been shown previously that the rIGS marker provides an excellent resolution of sub-clade structures within the PARB clade [12,13]. The identity of phylogenetic tree topologies generated from the single rIGS marker with those based on an integrative phylogenomic approach that is further backed by phenotypic traits strongly supports the idea that rIGS-based topological distinctions and sub-clade delineations are not only technically solid, but also phylogenetically meaningful. In conclusion, the observed congruence with a phylogenomic dataset validates the phylogenetic signal within the rIGS marker, thereby justifying its use as a surrogate for more resource-intensive methods for species delineation within the *Metarhizium* PARB clade.

Mining of GenBank data revealed 115 cryptic descriptions of *M. hybridum* and 30 of *M. parapingshaense*. Expectedly, most identified *M. hybridum* and *M. parapingshaense* strains had previously been assigned to *M. anisopliae* and *M. pingshaense*, respectively, or had only been identified at the genus level. Analysis of related publications revealed that the delineation of *M. hybridum* from *M. anisopliae* had been anticipated in several studies without being explicitly claimed [4,10,32]. Interestingly, *Metarhizium anisopliae* strain E6, i.e., a prominent model for biocontrol, molecular entomopathogenicity, genomics and secretomics studies [33,34,35,36,37,38], was identified as *M. hybridum*; knowledge gathered using strain E6 should therefore be attributed to this new species.

With respect to geographic origins, revealed data sets were strongly biased towards the Western hemisphere for *M. hybridum* and to East Asia and Oceania for *M. parapingshaense.* These apparent distributions are fully in line with the fact that all previous explicit descriptions of *M. hybridum* were from Brazil and Argentina, as well as from Thailand and the Solomon Islands for *M. parapingshaense*. However, this geographic bias is not necessarily meaningful with respect to the global distribution of the new taxa, as results from several extensive regional studies from China, Brazil or Mexico that address fungal diversity in particular regionally important ecosystems dominate the dataset. Nevertheless, the data presented formally constitute the first descriptions of the new species from Canada, China, Colombia, Costa Rica, Cuba, Honduras, Mexico, New Zealand, the Philippines and the USA in the case of *M. hybridum,* as well as from China, India, Japan, the Philippines and South Korea for *M. parapingshaense*.

A similar bias as for geographic origin data was observed with respect to the most frequent substrate of isolation for *M. hybridum* (insects) and *M. parapingshaense* (soil), which could potentially be indicative of a predominantly entomopathogenic lifestyle of *M. hybridum* and a saprophytic lifestyle of *M. parapingshaense.* However, as for geographic origin data, the currently available data set does by no means allow us to rule out a sampling artifact.

The species *M. hybridum* has been described as characterized by genetic mixed ancestry, presumably resulting from a genomic hybridization event involving *M. anisopliae sensu stricto* and a second unidentified PARB clade species [25]. It might be tempting to speculate that the second hybridization partner could have been *M. humberi.* This species had not been considered in the study introducing *M. hybridum,* and the currently known apparent geographic distributions of both *M. humberi* (Brazil, Mexico) and *M. hybridum* strongly coincide. Moreover, at least in the rIGS-based phylogenetic trees, *M. hybridum* is organized between *M. anisopliae* and *M. humberi* as would be consistent with a scenario of recombination between different intergenic spacer elements coexisting in the same genome during the early post-hybridization stage prior to purifying genome reduction with paralogous rRNA operon homogenization. It goes without saying that this intriguing but highly speculative hypothesis will require further substantiation from comparative genomics studies.

The *Metarhizium* PARB clade consists of a tight cluster of species comprising numerous *Metarhizium* strains of high relevance for both fundamental research in entomopathogenicity and microbial control of insect pests. Fast and reliable species-level assignment of fungal isolates is highly solicited across this group of fungi. Morphological and microscopic identification have clear limits with respect to the discrimination of PARB clade species, and species-level identification relies on genetic marker sequence determination. However, as reasonably fast, reliable, and inexpensive DNA sequencing may not be readily available for laboratories in many parts of the world, a sequencing-independent molecular approach can be expected to meet the needs of numerous researchers in the field.

Using the intergenic spacer (rIGS) sequence of the ribosomal RNA operon clusters of the *Metarhizium* genome as a target sequence, a species-discriminating diagnostic PCR tool had previously been established and demonstrated to discriminate between PARB clade species. The rationale behind this tool and the corresponding application strategy have been discussed previously [13]. The new diagnostic primer pairs designed and validated in the present study extend the existing approach, enabling the sequencing-independent identification of the newly delineated species *M. anisopliae, M. hybridum, M. pingshaense and M. parapingshaense*.

With respect to species identification within *M. anisopliae sensu lato,* the diagnostic PCR tool, as it currently stands, is subject to the highly significant *caveat* that—due to the inaccessibility of *M. humberi* reference strains—functionality of primer pairs for the delineation of *M. anisopliae* or *M. hybridum* from *M. humberi* cannot be tested experimentally, but has to rely on in silico analyses of published sequence data. This means that in diagnostic practice, there is a risk of generating false positives for *M. anisopliae sensu stricto* or *M. hybridum* from a *M. humberi* template DNA. Therefore, the correctness of PCR-based identification in *M. anisopliae sensu lato* should be confirmed by rIGS-ID800 sequence determination, as has been done in the present study. This is, of course, only a second-best solution for a diagnostic tool designed to enable sequencing-independent species-level identification.

## 5. Conclusions

Phylogenetic reconstruction based on ribosomal intergenic spacer (rIGS) sequences was employed to corroborate the previous introduction of the two new *Metarhizium* species, *M. hybridum* and *M. parapingshaense*, in demonstrating that these were not only correctly delineated from *M. anisopliae sensu stricto* and *M. pingshaense sensu stricto*, but also from all further species described in *M. anisopliae sensu lato* and *M. pingshaense sensu lato*, respectively. In turn, this confirmation of new species delineations derived from studies integrating whole genomes with phenotypic data lends strong support to the reliability of rIGS as a single-marker for species differentiation, at least in the *Metarhizium* PARB clade. A rIGS-based species-discriminating diagnostic PCR tool enabling sequencing-independent species identification has been functionally extended to these new *Metarhizium* species.

## Figures and Tables

**Table 1:** Comprehensive overview of cryptic descriptions of *Metarhizium hybridum* and *Metarhizium parapingshaense* identified in the GenBank database; detailed data, including GenBank accession numbers, were organized in Supplementary Table S3. Numbers in brackets indicate the number of strains or isolates identified.

Revised Taxonomic Assignment	Previous Taxonomic Assignment	Identification by Marker	Geographic Origin	Original Host/ Isolation Substrate
*Metarhizium hybridum* (115 strains/isolates)	*Metarhizium acridum* (1) *Metarhizium album* (1) *Metarhizium anisopliae* (76) *Metarhizium* sp. (37)	rIGS-ID800 (38) 5TEF (67) DUF895 (26) EF1A (10) RPB2 (13)	Argentina (2) Brazil (80) Canada (1) China (1) Colombia (2) Costa Rica (2) Cuba (5) Honduras (1) Mexico (12) New Zealand (1) Philippines (2) USA (1)	Insect (77) Plant (1) Soil (22)
*Metarhizium parapingshaense* (30 strains/isolates)	*Metarhizium anisopliae* (3) *Metarhizium pingshaense* (26) *Metarhizium* sp. (1)	rIGS-ID800 (2) 5TEF (6) DUF895 (23) EF1A (5) RPB2 (0)	China (16) India (5) Japan (6) Philippines (1) Solomon Islands (1) South Korea (1)	Insect (2) Soil (23) Water (1)

**Table 2:** Species-discriminating diagnostic PCR primer pairs used in this study.

Primer Designation	Nucleotide Sequence (5′ => 3′)	Expected Specificity	Approximate Product Size
manihyb-IDF2	GACACGCGTTGCGTTGT	*M. anisopliae* (mani-ID)	850 bp
mani-IDR2	ACTGCCATTCGCGCGGAG		
manihyb-IDF2	GACACGCGTTGCGTTGT	*M. hybridum* (mhyb-ID)	850 bp
mhyb-IDR2	GCCCTACCAAACTGCGAG		
mppi-IDF2	GTGGTTCTAGAGGGAAAAATCTGCCAAGTC	*M. parapingshaense* (mppi-ID)	250 bp
mppi-IDR2	CAAGTAAATCTACAAAGTCCAAAAATTG		
mpin-IDF2	ATCAATCGCAGCCTACCCGGTAAGTATAAG	*M. pingshaense* (mpin-ID)	700 bp
mpin-IDR2	GCCAAAATACTAGGAACTTGTATA		

## Data Availability

Sequence data analyzed in this study are publicly available from the GenBank database (https://www.ncbi.nlm.nih.gov, accessed on 18 February 2026) under nucleotide sequence accession numbers listed in Supplementary Tables S2 and S3 to this study.

## Supplementary Materials

The following supporting information can be downloaded at: https://www.mdpi.com/article/10.3390/jof12040272/s1, Figure S1: Bayesian Inference (BI) based phylogeny of *Metarhizium* fungi as reconstructed from complete rIGS sequences. Figure S2: Neighbor-Joining (NJ) phylogeny of *Metarhizium* fungi as reconstructed from rIGSID800 marker sequences. Figure S3: Neighbor-Joining (NJ) phylogeny of *Metarhizium* fungi as reconstructed from 5TEF marker sequences. Figure S4: Neighbor-Joining (NJ) phylogeny of *Metarhizium* fungi as reconstructed from DUF895 marker sequences. Figure S5: Neighbor-Joining (NJ) phylogeny of *Metarhizium* fungi as reconstructed from EF1A marker sequences. Figure S6: Neighbor-Joining (NJ) phylogeny of *Metarhizium* fungi as reconstructed from RPB2 marker sequences. Figure S7: Alignment of ribosomal intergenic spacer (rIGS) sequences used for the design of species-discriminating primers for *Metarhizium parapingshaense* and *Metarhizium pingshaense*. Figure S8: Alignment of ribosomal intergenic spacer (rIGS) sequences used for the design of species-discriminating primers for *Metarhizium hybridum* and *Metarhizium anisopliae*. Figure S9: Agarose gel electrophoresis of diagnostic PCRs using species-discriminating primer pairs. Table S1: *Metarhizium* strains and marker sequences referred to in this study. Table S2: Preparative PCR primers used and reaction-specific parameters applied in this study. Table S3: Cryptic descriptions of *M. hybridum* and *M. parapingshaense* identified in the GenBank database.
