# Supplementary material for: Species-Discriminating Diagnostic PCR, Ribosomal Intergenic Spacer-Based Single-Marker Taxonomy and Cryptic Descriptions of the Fungal Entomopathogens Metarhizium hybridum and Metarhizium parapingshaense
Source: J Fungi (Basel). 2026 Apr 9;12(4):272. doi: 10.3390/jof12040272 (PMC13117108; doi:10.3390/jof12040272)
Supplement: Supplementary file 1 [file jof-12-00272-s001.zip › Suppl Figure S1.pdf]

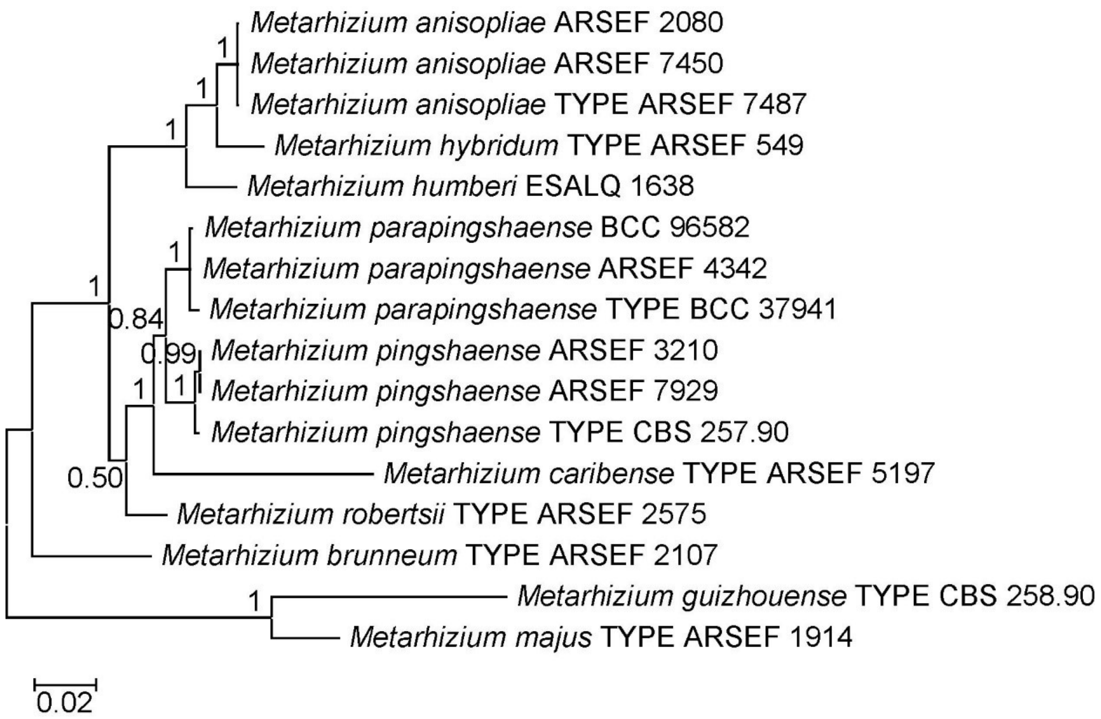

Supplementary Figure S1. Bayesian Inference (BI) based phylogeny of *Metarhizium* fungi as reconstructed from complete ribosomal intergenic spacer (rIGS) sequences. Terminal branches are labelled by genus, species and strain designations; "TYPE" denotes the nomenclatural type strain of a species. Numbers on branches indicate posterior probability (pp) values. The size bar indicates the number of expected substitutions per site. The orthologous sequences from the *M. majus* and *M. guizhouense* type strains were used as outgroup.
