# Supplementary material for: Species-Discriminating Diagnostic PCR, Ribosomal Intergenic Spacer-Based Single-Marker Taxonomy and Cryptic Descriptions of the Fungal Entomopathogens Metarhizium hybridum and Metarhizium parapingshaense
Source: J Fungi (Basel). 2026 Apr 9;12(4):272. doi: 10.3390/jof12040272 (PMC13117108; doi:10.3390/jof12040272)
Supplement: Supplementary file 1 [file jof-12-00272-s001.zip › Suppl Figure S6.pdf]

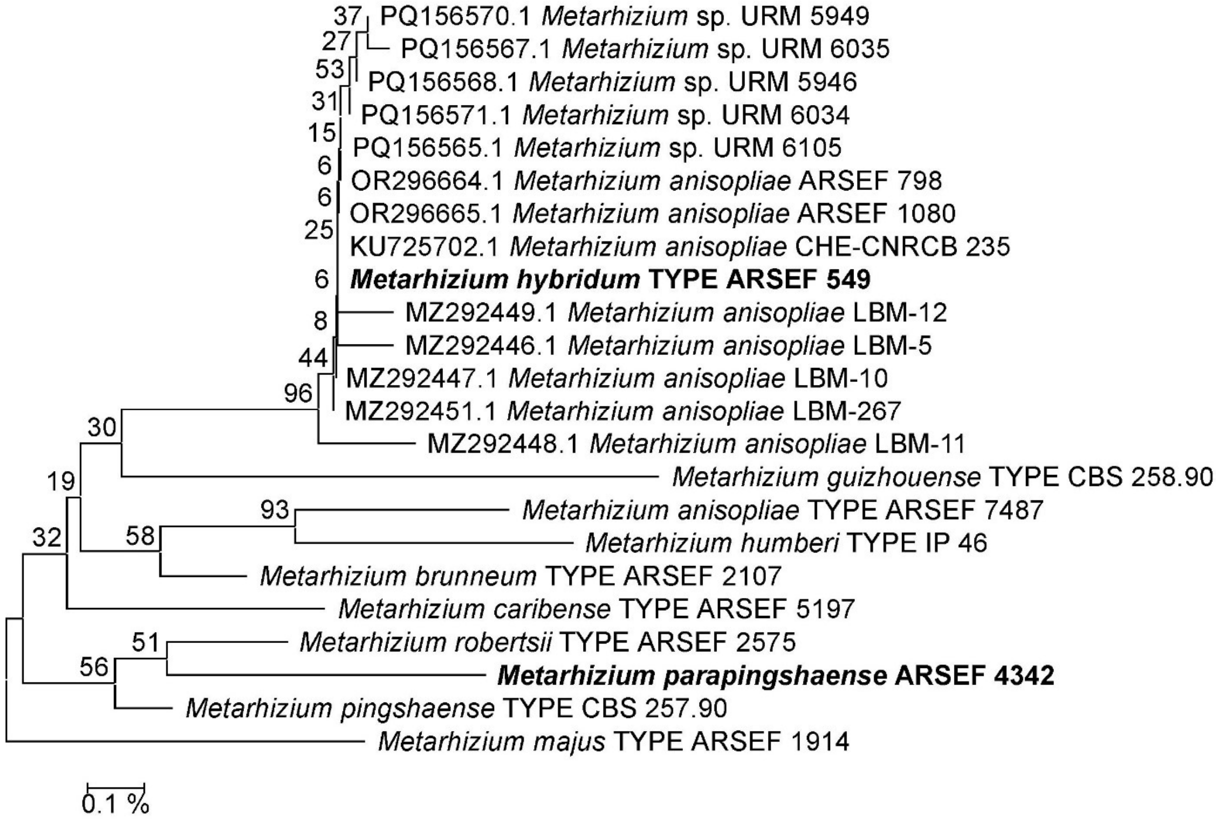

Supplementary Figure S6. Neighbor joining (NJ) phylogeny of *Metarhizium* fungi as reconstructed from RPB2 marker sequences. Terminal branches are labelled by genus, species and strain designations; "TYPE" denotes the nomenclatural type strain of a species. *M. hybridum* and *M. parapingshaense* reference strains are displayed in bold face. GenBank accession numbers are indicated for cryptic descriptions identified in the GenBank database. Numbers on branches indicate bootstrap support percentages. The size bar corresponds to 0.1% sequence divergence. The orthologous sequence from the *M. majus* type strain was used as outgroup.
