# Supplementary material for: Species-Discriminating Diagnostic PCR, Ribosomal Intergenic Spacer-Based Single-Marker Taxonomy and Cryptic Descriptions of the Fungal Entomopathogens Metarhizium hybridum and Metarhizium parapingshaense
Source: J Fungi (Basel). 2026 Apr 9;12(4):272. doi: 10.3390/jof12040272 (PMC13117108; doi:10.3390/jof12040272)
Supplement: Supplementary file 1 [file jof-12-00272-s001.zip › Suppl Figure S7.pdf]

mppi-IDF2	--GT-GGTTCTAGAGGGAAAAATCTGCCAAGT	100
mppi-IDR2 anti	--	100
mpin-IDF2	--	100
mpin-IDR2 anti	--	100
M. parapingshaense TYPE BCC 3794	GCGGGGCGGGTGTTGGGGTTAAAGCCACCAA-GTAGTGGTTGCAGGCTTGAGGAGAGCCGCCGAG---TGGT-GGTTCTAGAGGGAAAGATCTGCCAAGT	100
M. parapingshaense BCC 96582	GCGGGGCGGGTGTTGGGGTTAAAGCCACCAA-GTAGTGGTTGCAGGCTTGAGGAGAGCCGCCGAG---TGGT-GGTTCTAGAGGGAAAAATCTGCCAAGT	100
M. parapingshaense ARSEF 4342	GCGGGGCGGGTGTTGGGGTTAAAGCCACCAA-GTAGTGGTTGCAGGCTTGAGGAGAGCCGCCGAG---TGGT-GGTTCTAGAGGGAAAAATCTGCCAAGT	100
M. pingshaense TYPE CBS 257.90	GCGGGGCGGGTGTTGGGGTTAAAGCCACCAAAGTAGTGGTTGCAGGCTTGAGGAGAGCCGCCGAGCGGTGGTTGGTTCTAGAGGGGAAAATCTGCCAAGT	100
M. pingshaense ARSEF 3210	GCGGGGCGGGTGTTGGGGTTAAAGCCGCCAAAGTAGTGGTTGCAGGCTTGAGGAGAGCCGCCGAGCGGTGGTTGGTTCTAGAGGGGAAAATCTGCCAAGT	100
M. pingshaense ARSEF 7929	GCGGGGCGGGTGTTGGGGTTAAAGCCGCCAAAGTAGTGGTTGCAGGCTTGAGGAGAGCCGCCGAGCGGTGGTTGGTTCTAGAGGGGAAAATCTGCCAAGT	100
mppi-IDF2	C---	200
mppi-IDR2 anti	--	200
mpin-IDF2	--	200
mpin-IDR2 anti	--	200
M. parapingshaense TYPE BCC 3794	CCAAAGGTCAGGCAAGCGAAAGGTTCACCGAGTCGGAAAGTCGGGCGAGTAAAAAATTTACCAAGTCCAAAATACTGCCGGCAACATACCCGGGACTCCG	200
M. parapingshaense BCC 96582	CCAAAGGTCAGGCAAGCGAAAGGTTCACCGAGTCGGAAAGTCGGGCGAGTAAAAAATTTACCAAGTCCAAAATACTGCCGGCAACATACCCGGGACTCCG	200
M. parapingshaense ARSEF 4342	CCAAAGGTCAGGCAAGCGAAAGGTTCACCGAGTCGGAAAGTCGGGCGAGTAAAAAATTTACCAAGTCCAAAATACTGCCGGCAACATACCCGGGACTCCG	200
M. pingshaense TYPE CBS 257.90	TCAAAGGTCAGGCAAGCGAAAGGTTTACCGAGTCGGAAGGTTGGGCGAGTAAAAAATTTACCAAGTCCAAAATACTGCCGGCAACATACCCGGGACTCCG	200
M. pingshaense ARSEF 3210	TCAAAGGTCAGGCAAGCGAAAGGTTTACCGAGTCGGAAGGTTGGGCGAGTAAAAAATTTACCAAGTCCAAAATACTGCCGGCAACATACCCGGGACTCCG	200
M. pingshaense ARSEF 7929	TCAAAGGTCAGGCAAGCGAAAGGTTTACCGAGTCGGAAGGTTGGGCGAGTAAAAAATTTACCAAGTCCAAAATACTGCCGGCAACATACCCGGGACTCCG	200
mppi-IDF2	--	300
mppi-IDR2 anti	--CAATTTTTGG	300
mpin-IDF2	--	300
mpin-IDR2 anti	--	300
M. parapingshaense TYPE BCC 3794	AGTAAGTCGGGGAAGAAGTTGGCGGATTTTTTCCCAACCAACTCAACTT--GACCAATTTACCTGCCCGAGCTCACGGGTAGGCTGCGGTCAATTTTTGG	300
M. parapingshaense BCC 96582	AGTAAGTCGGGGAAGAAGTTGGCGGATTTTTTCCCAACCAACTCAACTT--GACCAATTTACCTGCCCGAGCTCACGGGTAGGCTGCGGTCAATTTTTGG	300
M. parapingshaense ARSEF 4342	AGTAAGTCGGGGAAGAAGTTGGCGGATTTTTTCCCAACCAACTCAACTT--GACCAATTTACCTGCCCGAGCTCACGGGTAGGCTGCGGTCAATTTTTGG	300
M. pingshaense TYPE CBS 257.90	AGTAAGTCGGGGAAGAAGTTGGCGGATTTTTTCCCAACCAACTCAACTTTTGACCAATTTACCTGCCCGAGCTCACGGGTAGGCTGCGGTTTATTTTTGG	300
M. pingshaense ARSEF 3210	AGTAAGTCGGGGAAGAAGTTGGCGGATTTTTTCCCAACCAACTCAACTTTTGACCAATTTACCTGCCCGAGCTCACGGGTAGGCTGCGGTTTATTTTTGG	300
M. pingshaense ARSEF 7929	AGTAAGTCGGGGAAGAAGTTGGCGGATTTTTTCCCAACCAACTCAACTTTTGACCAATTTACCTGCCCGAGCTCACGGGTAGGCTGCGGTTTATTTTTGG	300

mppi-IDF2	--	400
mppi-IDR2 anti	ACTTTGTAGATTTACTTG--	400
mpin-IDF2	--	400
mpin-IDR2 anti	--	400
M. parapingshaense TYPE BCC 3794	ACTTTGTAGATTTACTTGAATCAATTCACAAGAAGTTACCTGCCGAATTACCTGGGCTCCCGGGTAGGCTATAGTCAACTTTTGGACTTCCGACTATAAC	400
M. parapingshaense BCC 96582	ACTTTGTAGATTTACTTGAATCAATTCACAAGAAGTTACCTGCCGAATTACCTGGGCTCCCGGGTAGGCTATAGTCAACTTTTGGACTTCCGACTATAAC	400
M. parapingshaense ARSEF 4342	ACTTTGTAGATTTACTTGAATCAATTCACAAGAAGTTACCTGCCGAATTACCTGGGCTCCCGGGTAGGCTATAGTCAACTTTTGGACTTCCGACTATAAC	400
M. pingshaense TYPE CBS 257.90	ACTTTGTAGATTTGCTTGAATCAATTTACAGGAAGTTGCCTGCCGAATTACCTGGGCTCCCGGGTAGGCTATAGTTAACTTTTGGACTTTCGACTATAAC	400
M. pingshaense ARSEF 3210	ACTTTGTAGATTTGCTTGAATCAATTTACAGGAAGTTGCCTGCCGAATTACCTGGGCTCCCGGGTAGGCTATAGTTAACTTTTGGACTTTCGACTATAAC	400
M. pingshaense ARSEF 7929	ACTTTGTAGATTTGCTTGAATCAATTTACAGGAAGTTGCCTGCCGAATTACCTGGGCTCCCGGGTAGGCTATAGTTAACTTTTGGACTTTCGACTATAAC	400

mppi-IDF2	--	500
mppi-IDR2 anti	--	500
mpin-IDF2	--	500
mpin-IDR2 anti	--	500
M. parapingshaense TYPE BCC 3794	CTGGGATTTTCCCAACTATA--GCTCACGGGTAGGTTAGTTGTGATTTTTGGACTTGGTGAAATTTCTACTTTCCTGCCAAGTCTAGCCTACCCGGGAGC	500
M. parapingshaense BCC 96582	CTGGGATTTTCCCAACTATA--GCTCACGGGTAGGTTAGTTGTGATTTTTGGACTTGGTGAAATTTCTACTTTCCTGCCAAGTCTAGCCTACCCGGGAGC	500
M. parapingshaense ARSEF 4342	CTGGGATTTTCCCAACTATA--GCTCACGGGTAGGTTAGTTGTGATTTTTGGACTTGGTGAAATTTCTACTTTCCTGCCAAGTCTAGCCTACCCGGGAGC	500
M. pingshaense TYPE CBS 257.90	CTGGGATTTTCCCAACTATA--GCTCACGGGTAGGTTAGTTGTGATTTTTGGAC--GGTGAAATTTCTACTTTCCTGCCAAGTCTAGCCTACCCGGGAGC	500
M. pingshaense ARSEF 3210	CTGGGATTTTCCCAACTATATAGCTCACGGGTAGGTTAGTTGTGATTTTTGGACTTGGTGAAATTTCTACTTTCCTGCCAAGTCTAGCCTACCCGGGAGC	500
M. pingshaense ARSEF 7929	CTGGGATTTTCCCAACTATATAGCTCACGGGTAGGTTAGTTGTGATTTTTGGACTTGGTGAAATTTCTACTTTCCTGCCAAGTCTAGCCTACCCGGGAGC	500

mppi-IDF2	--	600
mppi-IDR2 anti	--	600
mpin-IDF2	--ATCAATCGCAGCCTAC	600
mpin-IDR2 anti	--	600
M. parapingshaense TYPE BCC 3794	CCAGGCAAGTCGGGCAGGTAAATTCCGCCAACTCGGCAGGAAACTAATTGCAAATCAATTCAAGCAAATATGCCAAGTCCACAAATTAATCGCAGCCTAC	600
M. parapingshaense BCC 96582	CCAGGCAAGTCGGGCAGGTAAATTCCGCCAACTCGGCAGGAAACTAATTGCAAATCAATTCAAGCAAATATGCCAAGTCCACAAATTAATCGCAGCCTAC	600
M. parapingshaense ARSEF 4342	CCAGGCAAGTCGGGCAGGTAAATTCCGCCAACTCGGCAGGAAACTAATTGCAAATCAATTCAAGCAAATATGCCAAGTCCACAAATTAATCGCAGCCTAC	600
M. pingshaense TYPE CBS 257.90	CCAGGCAAGTCGGGCAGGTAAATTCCGCCAACTCGGCAGGAAACTAATTGCAAATCAATTCAAGCAAATATGCCAAGTCCACAAATCAATCGCAGCCTAC	600
M. pingshaense ARSEF 3210	CCAGGCAAGTCGGGCAGGTAAATTCCGCCAACTCGGCAGGAAACTAATTGCAAATCAATTCAAGCAAATATGCCAAGTCCACAAATCAATCGCAGCCTAC	600
M. pingshaense ARSEF 7929	CCAGGCAAGTCGGGCAGGTAAATTCCGCCAACTCGGCAGGAAACTAATTGCAAATCAATTCAAGCAAATATGCCAAGTCCACAAATCAATCGCAGCCTAC	600

mppi-IDF2	--	700
mppi-IDR2 anti	--	700
mpin-IDF2	CCGGTAAGTATAAG--	700
mpin-IDR2 anti	--	700
M. parapingshaense TYPE BCC 3794	CCGGTAAGCCTGTGTAATT------------------------TTTTGCGCAAAATAAAAAATAAGCTATAAAAGCCTGGTAGCGGGGGCGGGCTCTTAA	700
M. parapingshaense BCC 96582	CCGGTAAGCCTGTGTAATT------------------------TTTTGCGGAAAATAAAAAATAAGCTATAAAAGCCTGGTAGCGGAGGCGGGCTCTTAA	700
M. parapingshaense ARSEF 4342	CCGGTAAGCCTGTGTAATT------------------------TTTTGCGGAAAATAAAAAATAAGCTATAAAAGCCTGGTAGCGGAGGCGGGCTCCTAA	700
M. pingshaense TYPE CBS 257.90	CCGGTAAGTATAAGTTGATTTTTTTTT-ATATATATAAATAAATTCTGCGGAAAATAAAAAATAAGCTATAAAGGCCTGGTAGCGGAGGCGAGCTCTTAA	700
M. pingshaense ARSEF 3210	CCGGTAAGTATAAGTTGATTTTTTTTTTATATATATAAATAAATTTTGCGGAAAATAAAAAATAAGCTATAAAGGCCTGGTAGCGGAGGCGAGCTCTTAA	700
M. pingshaense ARSEF 7929	CCGGTAAGTATAAGTTGATTTTTTTTTTATATATATAAATAAATTTTGCGGAAAATAAAAAATAAGCTATAAAGGCCTGGTAGCGGAGGCGAGCTCTTAA	700

mppi-IDF2	--	800
mppi-IDR2 anti	--	800
mpin-IDF2	--	800
mpin-IDR2 anti	--	800
M. parapingshaense TYPE BCC 3794	GGTAGTTGGTGGGTATATAAGAGGGAGGGCTGGACACGCGTTGCGTCGCGTATTTCCACCGCACCCAATACTTTTAGCTTTAGGGTAGGCTGCTTGTTTA	800
M. parapingshaense BCC 96582	GGTAGTTGGTGGGTATATAAGAGGGAGGGCTGGACACGCGTTGCGTCGCGTATTTCCACCGCACCCAATACTTTTAGCTTTAGGGTAGGCTGCTTGTTTA	800
M. parapingshaense ARSEF 4342	GGTAGTTGGTGGGTATATAAGAGGGAGGGCTGGACACGCGTTGCGTCGCGTATTTCCACCGCACCCAATACTTTTAGCTTTAGGGTAGGCTGCTTGTTTA	800
M. pingshaense TYPE CBS 257.90	GGTAGTGGGTGGGTATATAAGAGGGAGGGCTGGACACGCGTTGCGCCGCGTATTTCCACCGCACCCAATACTTTTAGCTTTAGGGTAGGCTGCTTGTTTA	800
M. pingshaense ARSEF 3210	GGTAGTGGGTGGGTATATAAGAGGGAGGGCTGGACACGCGTTGCGCCGCGTATTTCCACCGCACCCAATACTTTTAGCTTTAGGGTAGGCTGCTTGTTTA	800
M. pingshaense ARSEF 7929	GGTAGTGGGTGGGTATATAAGAGGGAGGGCTGGACACGCGTTGCGCCGCGTATTTCCACCGCACCCAATACTTTTAGCTTTAGGGTAGGCTGCTTGTTTA	800

mppi-IDF2	--	900
mppi-IDR2 anti	--	900
mpin-IDF2	--	900
mpin-IDR2 anti	--	900
M. parapingshaense TYPE BCC 3794	GAGGCGTGCTGAATTAAATGGTCTCTCAAGTGAGGGGATTTCTCTGCTGGCAGTTGCTTGTGATCCGGGAGTCCGTGGCGGTAAAGTCAACTGTAAGGCT	900
M. parapingshaense BCC 96582	GAGGCGTGCTGAATTAAATGGTCTCTCAAGTGAGGGGATTTCTCTGCTGGCAGTTGCTTGTGATCCGGGAGTCCGTGGCGGTAAAGTCAACTGTAAGGCT	900
M. parapingshaense ARSEF 4342	GAGGCGTGCTGAATTAAATGGTCTCTCAAGTGAGGGGATTTCTCTGCTGGCAGTTGCTTGTGATCCGGGAGTCCGTGGCGGTAAAGTCAACTGTAAGGCT	900
M. pingshaense TYPE CBS 257.90	GAGGCGTGCTGAATTAAATGGTCTCTCAAGTGAGGGGATTTCTCTGCTGGCAGTTGCTTGTGATCCGGGAGTCCGTGGCGGTAAAGTCAACTGTAAGGCT	900
M. pingshaense ARSEF 3210	GAGGCGTGCTGAATTAAATGGTCTCTCAAGTGAGGGGATTTCTCTGCTGGCAGTTGCTTGTGATCCGGGAGTCCGTGGCGGTAAAGTCAACTGTAAGGCT	900
M. pingshaense ARSEF 7929	GAGGCGTGCTGAATTAAATGGTCTCTCAAGTGAGGGGATTTCTCTGCTGGCAGTTGCTTGTGATCCGGGAGTCCGTGGCGGTAAAGTCAACTGTAAGGCT	900

mppi-IDF2	--	1000
mppi-IDR2 anti	--	1000
mpin-IDF2	--	1000
mpin-IDR2 anti	--	1000
M. parapingshaense TYPE BCC 3794	TGTGTGTGTGCCGGGGCCCTGTAGGTCCCGACCAGGAACCCTACAGGTCACGTGCCGATATAATAAGACAGATAAGATATACGGCACGTGCTAATACACG	1000
M. parapingshaense BCC 96582	TGTGTGTGTGCCGGGGCCCTGTAGGTCCCGACCAGGAACCCTACAGGTCACGTGCCGATATAATAAGACAGATAAGATATACGGCACGTGCTAATACACG	1000
M. parapingshaense ARSEF 4342	TGTGTGTGTGCCGGGGCCCTGTAGGTCCCGACCAGGAACCCTACAGGTCACGTGCCGATATAATAAGACAGATAAGATATACGGCACGTGCTAATACACG	1000
M. pingshaense TYPE CBS 257.90	TGTGTGTGTGCCGGGGCCCTGTAGGTCCCGACCAGGAACCCTACAGGCCACGTGCCGATATAATAAGATAGATAAGGTATACGGCACGTGCTAATACACG	1000
M. pingshaense ARSEF 3210	TGTGTGTGTGCCGGGGCCCTGTAGGTCCCGACCAGGAACCCTACAGGTCACGTGCCGATACAATAAGATAGATAAGGTATACGGCACGTGCTAATACACG	1000
M. pingshaense ARSEF 7929	TGTGTGTGTGCCGGGGCCCTGTAGGTCCCGACCAGGAACCCTACAGGTCACGTGCCGATACAATAAGATAGATAAGGTATACGGCACGTGCTAATACACG	1000

mppi-IDF2	--	1100
mppi-IDR2 anti	--	1100
mpin-IDF2	--	1100
mpin-IDR2 anti	--	1100
M. parapingshaense TYPE BCC 3794	TGCTAATACACGTGCCAATACAAGTAACGATAAGATCAACAAGATACCTGCACGTGCCTAGTCACGGAGACTTACACGTGTCCCGCTGCCAAGATCCCCG	1100
M. parapingshaense BCC 96582	TGC------------CAATACAAGTAACGATAAGATCAACAAGATACCTGCACGTGCCTAGTCACGGAGACTTACACGTGTCCCGCTGCCAAGATCCCCG	1100
M. parapingshaense ARSEF 4342	TGC------------CAATACAAGTAACGATAAGATCAACAAGATACCTGCACGTGCCTAGTCACGGAGACTTACACGTGTCCCGCTGCCAAGATCCCCG	1100
M. pingshaense TYPE CBS 257.90	TGC------------CAATACAAGTAACGATAAGATCAACAAGATACCTGCACGTGCCTAGTCACGGAGACTTACACGTGTCCCGCTGCCAAGATCCCCG	1100
M. pingshaense ARSEF 3210	TGC------------CAATACAAGTAACGATAAGATCAACAAGATACCTGCACGTGCCTAGTCACGGAGACTTACACGTGTCCCGCTGCCAAGATCCCCG	1100
M. pingshaense ARSEF 7929	TGC------------CAATACAAGTAACGATAAGATCAACAAGATACCTGCACGTGCCTAGTCACGGAGACTTACACGTGTCCCGCTGCCAAGATCCCCG	1100

mppi-IDF2	--	1200
mppi-IDR2 anti	--	1200
mpin-IDF2	--	1200
mpin-IDR2 anti	--	1200
M. parapingshaense TYPE BCC 3794	AGATTGCCGACCTCGCCGACTACTCCTGGTGTAATGTGCCGGTGTAGTTATATATAATTTCTTACTAGTCTCAATAATAGTACACTCGTTTATCGCGATG	1200
M. parapingshaense BCC 96582	AGATTGCCGACCTCGCCGACTACTCCTGGTGTAATGTGCCGGTGTAGTTATATATAATTTCTTACTAGTCTCAATAATAGTACACTCGTTTATCGCGATG	1200
M. parapingshaense ARSEF 4342	AGATTGCCGACCTCGCCGACTACTCCTGGTGTAATGTGCCGGTGTAGTTATATATAATTTCTTACTAGTCTCAATAATAGTACACTCGTTTATCGCGATG	1200
M. pingshaense TYPE CBS 257.90	AGGTTGCCGACCTCGCCGACTACTCCTGGTGTAATGTGCCGGTGTAGTTATATATAATTTCTTACTAGTCTCAATAATAGTACACTCGTTTATCGCGATG	1200
M. pingshaense ARSEF 3210	AGGTTGCCGACCTCGCCGACTACTCCTGGTGTAATGTGCCGGTGTAGTTATATATAATTTCTTACTAGTCTCAATAATAGTACACTCGTTTATCGCGATG	1200
M. pingshaense ARSEF 7929	AGGTTGCCGACCTCGCCGACTACTCCTGGTGTAATGTGCCGGTGTAGTTATATATAATTTCTTACTAGTCTCAATAATAGTACACTCGTTTATCGCGATG	1200

mppi-IDF2	--	1300
mppi-IDR2 anti	--	1300
mpin-IDF2	--	1300
mpin-IDR2 anti	--TATACAAGTTCCTA	1300
M. parapingshaense TYPE BCC 3794	CACTCACTTGTG---------------------------------GTGGTGCACTTACTCTACAATAGTGCTGGTACTTGCGGTCATATACAAGTTCCTA	1300
M. parapingshaense BCC 96582	CACTCACTTGTG---------------------------------GTGGTGCACTTACTCTACAATAGTGCTGGTACTTGCGGTCATATACAAGTTCCTA	1300
M. parapingshaense ARSEF 4342	CACTCACTTGTG---------------------------------GTGGTGCACTTACTCTACAATAGTGCTGGTACTTGCGGTCATATACAAGTTCCTA	1300
M. pingshaense TYPE CBS 257.90	CACTCACTTGTGGTGCGGGTGTGCGGGTGTGCTGACGTACTTGTGGTGGTGCACTTACTCTACAATAGTGCTGGTACTTGCGGTCATATACAAGTTCCTA	1300
M. pingshaense ARSEF 3210	CACTCACTTGTGGTGCGGGTGTGCGGGTGTGCTGACGTACTTGTGGTGGTGCACTTACTCTACAATAGTGCTGGTACTTGCGGTCATATACAAGTTCCTA	1300
M. pingshaense ARSEF 7929	CACTCACTTGTGGTGCGGGTGTGCGGGTGTGCTGACGTACTTGTGGTGGTGCACTTACTCTACAATAGTGCTGGTACTTGCGGTCATATACAAGTTCCTA	1300

mppi-IDF2	--	1400
mppi-IDR2 anti	--	1400
mpin-IDF2	--	1400
mpin-IDR2 anti	GTATTTTGGC--	1400
M. parapingshaense TYPE BCC 3794	GTATTTTGGCGTAGTTGTGGTCGTGGTCGTGCAGTTACAGCAGTGTACTTACCCTGTAGTACTGCTGGCACCGGCCACCGTCGCGTGCACGCGGCTGGCG	1400
M. parapingshaense BCC 96582	GTATTTTGGCGTAGTTGTGGTCGTGGTCGTGCAGTTACAGCAGTGTACTTACCCTGTAGTACTGCTGGCACCGGCCACCGTCGCGTGCACGCGGCTGCCG	1400
M. parapingshaense ARSEF 4342	GTATTTTGGCGTAGTTGTGGTCGTGGTCGTGCAGTTACAGCAGTGTACTTACCCTGTAGTACTGCTGGCACCGGCCACCGTCGCGTGCACGCGGCTGCCG	1400
M. pingshaense TYPE CBS 257.90	GTATTTTGGCGTAGTTGTGGCCGTGGTCGTGCAGTTACAGCAGTGTACTTACCCTGTAGTACTGCTGGCACCGGCCACCGTCGCGTGCACGCGGCTGCCG	1400
M. pingshaense ARSEF 3210	GTATTTTGGCGTAGTTGTGGCCGTGGTCGTGCAGTTACAGCAGTGTACTTACCCTGTAGTACTGCTGGCACCGGCCACCGTCGCGTGCACGCGGCTGCCG	1400
M. pingshaense ARSEF 7929	GTATTTTGGCGTAGTTGTGGCCGTGGTCGTGCAGTTACAGCAGTGTACTTACCCTGTAGTACTGCTGGCACCGGCCACCGTCGCGTGCACGCGGCTGCCG	1400

mppi-IDF2	--	1500
mppi-IDR2 anti	--	1500
mpin-IDF2	--	1500
mpin-IDR2 anti	--	1500
M. parapingshaense TYPE BCC 3794	TCGGCCGTGGTAGAGGGTTACCCGGGACCACGGTGCATTTTTCCGTTTTTTCCATTTTTTCAAAATCGACGACCAAAAAGTTGGTAGGTCTGGGTTGCCT	1500
M. parapingshaense BCC 96582	TCGGCCGTGGTAGAGGGTTACCCGGGACCACGGTGCATTTTTCCGTTTTTTCCATTTTTTCAAAATCGACGACCAAAAAGTTGGTAGGTCTGGGTTGCCT	1500
M. parapingshaense ARSEF 4342	TCGGCCGTGGTAGAGGGTTACCCGGGACCACGGTGCATTTTTCCGTTTTTTCCATTTTTTCAAAATCGACGACCAAAAAGTTGGTAGGTCTGGGTTGCCT	1500
M. pingshaense TYPE CBS 257.90	TCGGCCGTGGTAGAGGGTTACCCGGGACCACGGTGCATTTTTCCGTTTTTTCCATTTTTTCAAAATCGACGACCAAAAAGTTGGTAGGTCTGGGTTGCCT	1500
M. pingshaense ARSEF 3210	TCGGCCGTGGTAGAGGGTTACCCGGGACCACGGTGCATTTTTCCGTTTTTTCCATTTTTTCAAAATCGACGACCAAAAAGTTGGTAGGTCTGGGTTGCCT	1500
M. pingshaense ARSEF 7929	TCGGCCGTGGTAGAGGGTTACCCGGGACCACGGTGCATTTTTCCGTTTTTTCCATTTTTTCAAAATCGACGACCAAAAAGTTGGTAGGTCTGGGTTGCCT	1500

mppi-IDF2	--	1600
mppi-IDR2 anti	--	1600
mpin-IDF2	--	1600
mpin-IDR2 anti	--	1600
M. parapingshaense TYPE BCC 3794	TGCTGGCCAATAGAGATTTGTATGGCGCTACTATAGGGTAGGTGTAGGGTAGGCTGTACCGGTACTTGCGGTCGCACACAAATCCCTAGCAGCCTGGAGT	1600
M. parapingshaense BCC 96582	TGCTGGCCAATAGAGATTTGTATGGCGCTACTATAGGGTAGGTGTAGGGTAGGCTGTACCGGTACTTGCGGTCGCACACAAATCCCTAGCAGCCTGGAGT	1600
M. parapingshaense ARSEF 4342	TGCTGGCCAATAGAGATTTGTATGGCGCTACTATAGGGTAGGTGTAGGGTAGGCTGTACCGGTACTTGCGGTCGCACACAAATCCCTAGCAGCCTGGAGT	1600
M. pingshaense TYPE CBS 257.90	TGCTGGCCAATAGAGATTTGTATGGCGCTACTATAGGGTAGGTATAGGGTAGGCTGTACCGGTACTTGCGGTCGCACACAAATCCCTAGCAGCCTGGAGT	1600
M. pingshaense ARSEF 3210	TGCTGGCCAATAGAGATTTGTATGGCGCTACTATAGGGTAGGTATAGGGTAGGCTGTACCGGTACTTGCGGTCGCACACAAATCCCTAGCAGCCTGGAGT	1600
M. pingshaense ARSEF 7929	TGCTGGCCAATAGAGATTTGTATGGCGCTACTATAGGGTAGGTATAGGGTAGGCTGTACCGGTACTTGCGGTCGCACACAAATCCCTAGCAGCCTGGAGT	1600

mppi-IDF2	---------------------	1621
mppi-IDR2 anti	---------------------	1621
mpin-IDF2	---------------------	1621
mpin-IDR2 anti	---------------------	1621
M. parapingshaense TYPE BCC 3794	ACCCCCGCGCGCGGGCAACTT	1621
M. parapingshaense BCC 96582	ACCCCCGCGCGCGGGCAACTT	1621
M. parapingshaense ARSEF 4342	ACCCCCGCGCGCGGGCAACTT	1621
M. pingshaense TYPE CBS 257.90	ACCCCCGCGCGCGGGCAACTT	1621
M. pingshaense ARSEF 3210	ACCCCCGCGCGCGGGCAACTT	1621
M. pingshaense ARSEF 7929	ACCCCCGCGCGCGGGCAACTT	1621

Supplementary Figure S7. Alignment of ribosomal intergenic spacer (rIGS) sequences used for the design of species-discriminating primers for *Metarhizium parapingshaense* and *Metarhizium pingshaense*. Diagnostic primer sequences are marked yellow.
