# Supplementary material for: Species-Discriminating Diagnostic PCR, Ribosomal Intergenic Spacer-Based Single-Marker Taxonomy and Cryptic Descriptions of the Fungal Entomopathogens Metarhizium hybridum and Metarhizium parapingshaense
Source: J Fungi (Basel). 2026 Apr 9;12(4):272. doi: 10.3390/jof12040272 (PMC13117108; doi:10.3390/jof12040272)
Supplement: Supplementary file 1 [file jof-12-00272-s001.zip › Suppl Figure S8.pdf]

```
manihyb-IDF2                    ----------------------------------------------------------------------------------------------------  100
mani-IDR2 anti                  ----------------------------------------------------------------------------------------------------  100
mhyb-IDR2 anti                  ----------------------------------------------------------------------------------------------------  100
M. hybridum TYPE ARSEF 549      GCGGGGCGGGTGTTGGGGTTAGAGCCACCAAGTAGTGGTTACAGGCTTGAGGAGAGCTGCCGAGTGGTGGTTCTAGAGGGAAAAGTCTGCCAGGTTCAAA  100
M. hybridum ARSEF 1080          GCGGGGCGGGTGTTGGGGTTAGAGCCACCAAGTAGTGGTTACAGGCTTGAGGAGAGCTGCCGAGTGGTGGTTCTAGAGGGAAAAGTCTGCCAGGTTCAAA  100
M. hybridum ARSEF 6347          GCGGGGCGGGTGTTGGGGTTAGAGCCACCAAGTAGTGGTTACAGGCTTGAGGAGAGCTGCCGAGTGGTGGTTCTAGAGGGAAAAGTCTGCCAGGTTCAAA  100
M. anisopliae TYPE ARSEF 7487 GCGGGGCGGGTGTTGGGGTTAAAGCCACCAAGTAGTGGTTACAGGCTTGAGGGGAGCTGCCGAGTGGTGGTTCTAGAGGGAAAAGTCTGCCAAGTTCAAA  100
M. anisopliae ARSEF 7450        GCGGGGCGGGTGTTGGGGTTAAAGCCACCAAGTAGTGGTTACAGGCTTGAGGGGAGCTGCCGAGTGGTGGTTCTAGAGGGAAAAGTCTGCCAAGTTCAAA  100
M. anisopliae ARSEF 2080        GCGGGGCGGGTGTTGGGGTTAAAGCCACCAAGTAGTGGTTACAGGCTTGAGGGGAGCTGCCGAGTGGTGGTTCTAGAGGGAAAAGTCTGCCAAGTTCAAA  100

manihyb-IDF2                    ----------------------------------------------------------------------------------------------------  200
mani-IDR2 anti                  ----------------------------------------------------------------------------------------------------  200
mhyb-IDR2 anti                  ----------------------------------------------------------------------------------------------------  200
M. hybridum TYPE ARSEF 549      GGTCCAGGCAAGCGAAAAGTTTACCAAGTC---------------------------------CAAAATATTGGCGGTAACCTACCCGGGACTCCGAGTA  200
M. hybridum ARSEF 1080          GGTCCAGGCAAGCGAAAAGTTTACCAAGTCGGAAGGTTGGGCGAGTAAAAAAATTACCAAGTCCAAAATATTGGCGGTAACCTACCCGGGACTCCGAGTA  200
M. hybridum ARSEF 6347          GGTCCAGGCAAGCGAAAAGTTTACCAAGTCGGAAGGTTGGGCGAGTAAAAAAATTACCAAGTCCAAAATATTGGCGGTAACCTACCCGGGACTCCGAGTA  200
M. anisopliae TYPE ARSEF 7487 GGTCCAGGCAAGCGAAAAGTTTACCAAGTCGGAAGGTTGGGCGAGTAAAAAAATTACCAAGTCCAAAATATTGGCGGTAACCTACCCGGGACTCCGAGTA  200
M. anisopliae ARSEF 7450        GGTCCAGGCAAGCGAAAAGTTTACCAAGTCGGAAGGTTGGGCGAGTAAAAAAATTACCAAGTCCAAAATATTGGCGGTAACCTACCCGGGACTCCGAGTA  200
M. anisopliae ARSEF 2080        GGTCCAGGCAAGCGAAAAGTTTACCAAGTCGGAAGGTTGGGCGAGTAAAAAAATTACCAAGTCCAAAATATTGGCGGTAACCTACCCGGGACTCCGAGTA  200

manihyb-IDF2                    ----------------------------------------------------------------------------------------------------  300
mani-IDR2 anti                  ----------------------------------------------------------------------------------------------------  300
mhyb-IDR2 anti                  ----------------------------------------------------------------------------------------------------  300
M. hybridum TYPE ARSEF 549      AGTCGGGGAAAAAGTTGGCGGATTTTCCCCCAACCAACTCAACTTTTGACCAATTTACCTGCCCGAGCTCACGGGTAGGCTGCGGTTTATTTTTGGACTT  300
M. hybridum ARSEF 1080          AGTCGGGGAAAAAGTTGGCGGATTTTTCCCCAACCAACTCAACTTTTGACCAATTTACCTGCCCGAGCTCACGGGTAGGCTGCGGTTTATTTTTGGACTT  300
M. hybridum ARSEF 6347          AGTCGGGGAAAAAGTTGGCGGATTTTTCCCCAACCAACTCAACTTTTGACCAATTTACCTGCCCGAGCTCACGGGTAGGCTGCGGTTTATTTTTGGACTT  300
M. anisopliae TYPE ARSEF 7487 AGTCGGGGAAAAAGTTGGCGGATTTTTTCCCAACCAACTCAACTTTTGACCAATTTACCTGCCCGAGCTCACGGGTAGGCTGCGGTTTATTTTTGGACTT  300
M. anisopliae ARSEF 7450        AGTCGGGGAAAAAGTTGGCGGATTTTTTCCCAACCAACTCAACTTTTGACCAATTTACCTGCCCGAGCTCACGGGTAGGCTGCGGTTTATTTTTGGACTT  300
M. anisopliae ARSEF 2080        AGTCGGGGAAAAAGTTGGCGGATTTTTTCCCAACCAACTCAACTTTTGACCAATTTACCTGCCCGAGCTCACGGGTAGGCTGCGGTTTATTTTTGGACTT  300

manihyb-IDF2                    ----------------------------------------------------------------------------------------------------  400
mani-IDR2 anti                  ----------------------------------------------------------------------------------------------------  400
mhyb-IDR2 anti                  ----------------------------------------------------------------------------------------------------  400
M. hybridum TYPE ARSEF 549      TGTAGATTTACTTGAATCAATTCACGGGAAGTTACCTGCCGAATTACCTGGGCTCCCGGGTAGGCTATAGTCAACTTTTGGACTTGCGACTATAACCTGG  400
M. hybridum ARSEF 1080          TGTAGATTTACTTGAATCAATTCACGGGAAGTTACCTGCCGAATTACCTGGGCTCCCGGGTAGGCTATAGTCAACTTTTGGACTTGCGACTATAACCTGG  400
M. hybridum ARSEF 6347          TGTAGATTTACTTGAATCAATTCACGGGAAGTTACCTGCCGAATTACCTGGGCTCCCGGGTAGGCTATAGTCAACTTTTGGACTTGCGACTATAACCTGG  400
M. anisopliae TYPE ARSEF 7487 TGTAGATTTACTTGAATCAATTCACGGGAAGTTACCTGCCGAATTACCTGGGCTCCCGGGTAGGCTATAGTTAACTTTTGGACTTGCGACTATAACCTGG  400
M. anisopliae ARSEF 7450        TGTAGATTTACTTGAATCAATTCACGGGAAGTTACCTGCCGAATTACCTGGGCTCCCGGGTAGGCTATAGTTAACTTTTGGACTTGCGACTATAACCTGG  400
M. anisopliae ARSEF 2080        TGTAGATTTACTTGAATCAATTCACGGGAAGTTACCTGCCGAATTACCTGGGCTCCCGGGTAGGCTATAGTTAACTTTTGGACTTGCGACTATAACCTGG  400
```

```
manihyb-IDF2                   ---------------------------------------------------------------------------------------------------- 500
mani-IDR2 anti                 ---------------------------------------------------------------------------------------------------- 500
mhyb-IDR2 anti                 ---------------------------------------------------------------------------------------------------- 500
M. hybridum TYPE ARSEF 549     GATTTTCCCAACCAGAGCTCACGGGTAGGCTAGTTGTGATTTTTGGACTTGGTGAAATTTCTACTTTCCTGCCAAGTCTAGCCTACCCGGGAGCCCAGGC 500
M. hybridum ARSEF 1080         GATTTTCCCAACCAGAGCTCACGGGTAGGCTAGTTGTGATTTTTGGACTTGGTGAAATTTCTACTTTCCTGCCAAGTCTAGCCTACCCGGGAGCCCAGGC 500
M. hybridum ARSEF 6347         GATTTTCCCAACCAGAGCTCACGGGTAGGCTAGTTGTGATTTTTGGACTTGGTGAAATTTCTACTTTCCTGCCAAGTCTAGCCTACCCGGGAGCCCAGGC 500
M. anisopliae TYPE ARSEF 7487 GATTTTCCCAACCATAGCTCACGGGTAGGCTAGTTGTGATTTTTGGACTTGGTGAAATTTCTACTTTCCTGCCAAGTCTAGCCTACCCGGGAGCCCAGGC 500
M. anisopliae ARSEF 7450      GATTTTCCCAACCATAGCTCACGGGTAGGCTAGTTGTGATTTTTGGACTTGGTGAAATTTCTACTTTCCTGCCAAGTCTAGCCTACCCGGGAGCCCAGGC 500
M. anisopliae ARSEF 2080      GATTTTCCCAACCATAGCTCACGGGTAGGCTAGTTGTGATTTTTGGACTTGGTGAAATTTCTACTTTCCTGCCAAGTCTAGCCTACCCGGGAGCCCAGGC 500

manihyb-IDF2                   ---------------------------------------------------------------------------------------------------- 600
mani-IDR2 anti                 ---------------------------------------------------------------------------------------------------- 600
mhyb-IDR2 anti                 ---------------------------------------------------------------------------------------------------- 600
M. hybridum TYPE ARSEF 549     AAGTCGGGCAGGTAAATTCCGCCAACTCGGCAGGGAACTAATTGCAAATCAATTCGAGCAAATGTGCCAAGTCCACAAATTAATCGTAGCCTACCCGGTA 600
M. hybridum ARSEF 1080         AAGTCGGGCAGGTAAATTCCGCCAACTCGGCAGGGAACTAATTGCAAATCAATTCGAGCAAATGTGCCAAGTCCACAAATTAATCGTAGCCTACCCGGTA 600
M. hybridum ARSEF 6347         AAGTCGGGCAGGTAAATTCCGCCAACTCGGCAGGGAACTAATTGCAAATCAATTCGAGCAAATGTGCCAAGTCCACAAATTAATCGTAGCCTACCCGGTA 600
M. anisopliae TYPE ARSEF 7487 AAGTCGGGCAGGTAAATTCCGCCAACTCGGCAGGGAACTAATTGCAAATCAATTCGAGCAAATATGCCAAGTCCACAAATGAATCGCAGCCTACCCGGTA 600
M. anisopliae ARSEF 7450      AAGTCGGGCAGGTAAATTCCGCCAACTCGGCAGGGAACTAATTGCAAATCAATTCGAGCAAATATGCCAAGTCCACAAATGAATCGCAGCCTACCCGGTA 600
M. anisopliae ARSEF 2080      AAGTCGGGCAGGTAAATTCCGCCAACTCGGCAGGGAACTAATTGCAAATCAATTCGAGCAAATATGCCAAGTCCACAAATGAATCGCAGCCTACCCGGTA 600

manihyb-IDF2                   ---------------------------------------------------------------------------------------------------- 700
mani-IDR2 anti                 ---------------------------------------------------------------------------------------------------- 700
mhyb-IDR2 anti                 ---------------------------------------------------------------------------------------------------- 700
M. hybridum TYPE ARSEF 549     GGTAAGCCTGTGTAGTTTCCGTGTAATTTTCCCGCAAAGACCAAAAAGTCAGGTGTTTTAGTATTTAATTTATATAGGCGTGAGTTGATTTTTTTGTTTT 700
M. hybridum ARSEF 1080         GGTAAGCCTGTGTAGTTTCCGTGTAATTTTCCCGCAAAGACCAAAAAGTCAGGTGTTTTAGTATTTAATTTATATAGGCGTGAGTTGATTTTTTTGTTTT 700
M. hybridum ARSEF 6347         GGTAAGCCTGTGTAGTTTCCGTGTAATTTTCCCGCAAAGACCAAAAAGTCAGGTGTTTTAGTATTTAATTTATATAGGCGTGAGTTGATTTTTTTGTTTT 700
M. anisopliae TYPE ARSEF 7487 GGTAAGCCTGTGTAGTTTCCGTGTAATTTTCCCGTAAAGACCAAAAAGTCAGGTGTTTTAGTATTTAATTTATATAGGCGTGAGTTGATTTTTTTGTTTT 700
M. anisopliae ARSEF 7450      GGTAAGCCTGTGTAGTTTCCGTGTAATTTTCCCGTAAAGACCAAAAAGTCAGGTGTTTTAGTATTTAATTTATATAGGCGTGAGTTGATTTTTTTGTTTT 700
M. anisopliae ARSEF 2080      GGTAAGCCTGTGTAGTTTCCGTGTAATTTTCCCGTAAAGACCAAAAAGTCAGGTGTTTTAGTATTTAATTTATATAGGCGTGAGTTGATTTTTTTGTTTT 700

manihyb-IDF2                   ---------------------------------------------------------------------------------------------------- 800
mani-IDR2 anti                 ---------------------------------------------------------------------------------------------------- 800
mhyb-IDR2 anti                 ---------------------------------------------------------------------------------------------------- 800
M. hybridum TYPE ARSEF 549     TTTATATTAATAAATTTTGCGGAAAATAAAAAATAAGCTATAAAAACCTGGTAACAAAGGCGGGCTTTTAAGGTAATTGGTGGGTATATAAGAGGGAGGG 800
M. hybridum ARSEF 1080         TTTATATTAATAAATTTTGCGGAAAATAAAAAATAAGCTATAAAAACCTGGTAACAAAGGCGGGCTTTTAAGGTAATTGGTGGGTATATAAGAGGGAGGG 800
M. hybridum ARSEF 6347         TTTATATTAATAAATTTTGCGGAAAATAAAAAATAAGCTATAAAAACCTGGTAACAAAGGCGGGCTTTTAAGGTAATTGGTGGGTATATAAGAGGGAGGG 800
M. anisopliae TYPE ARSEF 7487 TCTATATTAATAAATTTTGCGGAAAATAAAAAATAAGCTATAAAAACCTGGTAACAGAGGCGGGCCCTTAAGGTAATTGGTGGGTATATAAGAGGGAGGG 800
M. anisopliae ARSEF 7450      TCTATATTAATAAATTTTGCGGAAAATAAAAAATAAGCTATAAAAACCTGGTAACAGAGGCGGGCCCTTAAGGTAATTGGTGGGTATATAAGAGGGAGGG 800
M. anisopliae ARSEF 2080      TCTATATTAATAAATTTTGCGGAAAATAAAAAATAAGCTATAAAAACCTGGTAACAGAGGCGGGCCCTTAAGGTAATTGGTGGGTATATAAGAGGGAGGG 800
```

```
manihyb-IDF2                  ---GACACGCGTTGCGTTGT--------------------------------------------------------------------------------  900
mani-IDR2 anti                ----------------------------------------------------------------------------------------------------  900
mhyb-IDR2 anti                ----------------------------------------------------------------------------------------------------  900
M. hybridum TYPE ARSEF 549    CTGGACACGCGTTGCGTTGTGTATTTCTACCATACCCAATACTTTTAGCTTTAGGGTAGGCTGCTTGTTTAGAGGCGTGCTGAATTAAATGGTCTCTTGA  900
M. hybridum ARSEF 1080        CTGGACACGCGTTGCGTTGTGTATTTCTACCATACCCAATACTTTTAGCTTTAGGGTAGGCTGCTTGTTTAGAGGCGTGCTGAATTAAATGGTCTCTTGA  900
M. hybridum ARSEF 6347        CTGGACACGCGTTGCGTTGTGTATTTCTACCATACCCAATACTTTTAGCTTTAGGGTAGGCTGCTTGTTTAGAGGCGTGCTGAATTAAATGGTCTCTTGA  900
M. anisopliae TYPE ARSEF 7487 CTGGACACGCGTTGCGTTGTGTATTTCCACCACACCCAATACTTTTAGCTTTAGGGTAGGCTGCTTGTTCAGAGGCGTGCTGAATTAAATGGTCTCTTGA  900
M. anisopliae ARSEF 7450      CTGGACACGCGTTGCGTTGTGTATTTCCACCACACCCAATACTTTTAGCTTTAGGGTAGGCTGCTTGTTCAGAGGCGTGCTGAATTAAATGGTCTCTTGA  900
M. anisopliae ARSEF 2080      CTGGACACGCGTTGCGTTGTGTATTTCCACCACACCCAATACTTTTAGCTTTAGGGTAGGCTGCTTGTTCAGAGGCGTGCTGAATTAAATGGTCTCTTGA  900

manihyb-IDF2                  ---------------------------------------------------------------------------------------------------- 1000
mani-IDR2 anti                ---------------------------------------------------------------------------------------------------- 1000
mhyb-IDR2 anti                ---------------------------------------------------------------------------------------------------- 1000
M. hybridum TYPE ARSEF 549    GTGAGGGGATTTCTCTGCTGGCAGTTGCCTGTGATCCGGGAGTCCGTGGCGGTAAAGTCAACTGTAAGGCTTGTGTGTGTGCCGGGGCCCTGTAAGTCCC 1000
M. hybridum ARSEF 1080        GTGAGGGGATTTCTCTGCTGGCAGTTGCCTGTGATCCGGGAGTCCGTGGCGGTAAAGTCAACTGTAAGGCTTGTGTGTGTGCCGGGGCCCTGTAAGTCCC 1000
M. hybridum ARSEF 6347        GTGAGGGGATTTCTCTGCTGGCAGTTGCCTGTGATCCGGGAGTCCGTGGCGGTAAAGTCAACTGTAAGGCTTGTGTGTGTGCCGGGGCCCTGTAAGTCCC 1000
M. anisopliae TYPE ARSEF 7487 GTGAGGGGATTTCTCTGCTGGCAGTTGCCTGTGATCCGGGAGTCCGTGGCGGTAAAGTCAACTGTAAGGCTTGTGTGTGTGCCGGGGCCCTGTAAGTCCC 1000
M. anisopliae ARSEF 7450      GTGAGGGGATTTCTCTGCTGGCAGTTGCCTGTGATCCGGGAGTCCGTGGCGGTAAAGTCAACTGTAAGGCTTGTGTGTGTGCCGGGGCCCTGTAAGTCCC 1000
M. anisopliae ARSEF 2080      GTGAGGGGATTTCTCTGCTGGCAGTTGCCTGTGATCCGGGAGTCCGTGGCGGTAAAGTCAACTGTAAGGCTTGTGTGTGTGCCGGGGCCCTGTAAGTCCC 1000

manihyb-IDF2                  ---------------------------------------------------------------------------------------------------- 1100
mani-IDR2 anti                ---------------------------------------------------------------------------------------------------- 1100
mhyb-IDR2 anti                ---------------------------------------------------------------------------------------------------- 1100
M. hybridum TYPE ARSEF 549    GACCAGGAACCCTACAGGTTATGTGCCGATACAATAAGACAGATAAGATATGCGGCACGTGCTAGTACACGTGCCAATACAAGTGACGATAAGATCGATA 1100
M. hybridum ARSEF 1080        GACCAGGAACCCTACAGGTTATGTGCCGATACAATAAGACAGATAAGATATGCGGCACGTGCTAGTACACGTGCCAATACAAGTGACGATAAGATCGATA 1100
M. hybridum ARSEF 6347        GACCAGGAACCCTACAGGTTATGTGCCGATACAATAAGACAGATAAGATATGCGGCACGTGCTAGTACACGTGCCAATACAAGTGACGATAAGATCGATA 1100
M. anisopliae TYPE ARSEF 7487 GACCAGGAACCCTACAGGTCACGTGCCGATACAATAAGACAGATAAGATATGCGGCACGTGCTAGTACACGTGCCAATACAAGTGACGATAAGATCGATA 1100
M. anisopliae ARSEF 7450      GACCAGGAACCCTACAGGTCACGTGCCGATACAATAAGACAGATAAGATATGCGGCACGTGCTAGTACACGTGCCAATACAAGTGACGATAAGATCGATA 1100
M. anisopliae ARSEF 2080      GACCAGGAACCCTACAGGTCACGTGCCGATACAATAAGACAGATAAGATATGCGGCACGTGCTAGTACACGTGCCAATACAAGTGACGATAAGATCGATA 1100

manihyb-IDF2                  ---------------------------------------------------------------------------------------------------- 1200
mani-IDR2 anti                ---------------------------------------------------------------------------------------------------- 1200
mhyb-IDR2 anti                ---------------------------------------------------------------------------------------------------- 1200
M. hybridum TYPE ARSEF 549    AGATACCTGCACGTGCCTAGTCACGGAGACTTACACGTGTTCCGCTGCCAAGGTCCCCGAGGTCCCCGCGATTGCCGACCTCGCCGACTACTCCTGGTGT 1200
M. hybridum ARSEF 1080        AGATACCTGCACGTGCCTAGTCACGGAGACTTACACGTGTTCCGCTGCCAAGGTCCCCGAGGTCCCCGCGATTGCCGACCTCGCCGACTACTCCTGGTGT 1200
M. hybridum ARSEF 6347        AGATACCTGCACGTGCCTAGTCACGGAGACTTACACGTGTTCCGCTGCCAAGGTCCCCGAGGTCCCCGCGATTGCCGACCTCGCCGACTACTCCTGGTGT 1200
M. anisopliae TYPE ARSEF 7487 AGATACCTGCACGTGCCTAGTCACGGAGACTTACACGTGTTCCGCTGCCAAGGTCCCCGAGGTCCCCGCGATTGCCGACCTCGCCGACTACTCCTGGTGT 1200
M. anisopliae ARSEF 7450      AGATACCTGCACGTGCCTAGTCACGGAGACTTACACGTGTTCCGCTGCCAAGGTCCCCGAGGTCCCCGCGATTGCCGACCTCGCCGACTACTCCTGGTGT 1200
M. anisopliae ARSEF 2080      AGATACCTGCACGTGCCTAGTCACGGAGACTTACACGTGTTCCGCTGCCAAGGTCCCCGAGGTCCCCGCGATTGCCGACCTCGCCGACTACTCCTGGTGT 1200
```

manihyb-IDF2	--	1300
mani-IDR2 anti	--	1300
mhyb-IDR2 anti	--	1300
M. hybridum TYPE ARSEF 549	AATATGCCGGTGTAGTTGTGTATAATTTCTTACTAGTCTTAATAGTAGTACACTTGTTTATCGCGATACACTTACTTGTG--------GTGCGGCTGTGC	1300
M. hybridum ARSEF 1080	AATATGCCGGTGTAGTTGTGTATAATTTCTTACTAGTCTTAATAGTAGTACACTTGTTTATCGCGATACACTTACTTGTG--------GTGCGGCTGTGC	1300
M. hybridum ARSEF 6347	AATATGCCGGTGTAGTTGTGTATAATTTCTTACTAGTCTTAATAGTAGTACACTTGTTTATCGCGATACACTTACTTGTG--------GTGCGGCTGTGC	1300
M. anisopliae TYPE ARSEF 7487	AATATACCGGTGTAGTTGTATATAATTTCTTACTAGTCTTAATAGTAGTACACTTGTTTATCGCGATACACTTACTTGTGGTGCGGCTGTGCGGCTGTGC	1300
M. anisopliae ARSEF 7450	AATATACCGGTGTAGTTGTATATAATTTCTTACTAGTCTTAATAGTAGTACACTTGTTTATCGCGATACACTTACTTGTGGTGCGGCTGTGCGGCTGTGC	1300
M. anisopliae ARSEF 2080	AATATACCGGTGTAGTTGTATATAATTTCTTACTAGTCTTAATAGTAGTACACTTGTTTATCGCGATACACTTACTTGTGGTGCGGCTGTGCGGCTGTGC	1300
manihyb-IDF2	--	1400
mani-IDR2 anti	--	1400
mhyb-IDR2 anti	--	1400
M. hybridum TYPE ARSEF 549	GGCTGTGCTGATGTACTTGTAGTGGTGCACTTACTCTACAATAGTGCTGGTACTTACGGTCGCACACAAATCCCTAGTATTTTGGTGTAGTTGTGGTCGT	1400
M. hybridum ARSEF 1080	GGCTGTGCTGATGTACTTGTAGTGGTGCACTTACTCTACAATAGTGCTGGTACTTACGGTCGCACACAAATCCCTAGTATTTTGGTGTAGTTGTGGTCGT	1400
M. hybridum ARSEF 6347	GGCTGTGCTGATGTACTTGTAGTGGTGCACTTACTCTACAATAGTGCTGGTACTTACGGTCGCACACAAATCCCTAGTATTTTGGTGTAGTTGTGGTCGT	1400
M. anisopliae TYPE ARSEF 7487	GGCTGTGCTGACGTACTTGTAGTGGTGCACTTACTCTACAATAGTGCTGGTACTTACGGTCGCACACAAATCCCTAGTATTTTGGTGTAGTTGTGATCGT	1400
M. anisopliae ARSEF 7450	GGCTGTGCTGACGTACTTGTAGTGGTGCACTTACTCTACAATAGTGCTGGTACTTACGGTCGCACACAAATCCCTAGTATTTTGGTGTAGTTGTGATCGT	1400
M. anisopliae ARSEF 2080	GGCTGTGCTGACGTACTTGTAGTGGTGCACTTACTCTACAATAGTGCTGGTACTTACGGTCGCACACAAATCCCTAGTATTTTGGTGTAGTTGTGATCGT	1400
manihyb-IDF2	--	1500
mani-IDR2 anti	--	1500
mhyb-IDR2 anti	--	1500
M. hybridum TYPE ARSEF 549	GGTGGTGCAGTTACAGCAGTGCACTTACCCTGTAGTACTGCTGGCACCGGCCACCGTCGGCCGTGGCGGAGGGTTACCCGGGACCACAGTGCATTTTTCC	1500
M. hybridum ARSEF 1080	GGTGGTGCAGTTACACCAGTGCACTTACCCTGTAGTACTGCTGGCACCGGCCACCGTCGGCCGTGGCGGAGGGTTACCCGGGACCACAGTGCATTTTTCC	1500
M. hybridum ARSEF 6347	GGTGGTGCAGTTACAGCAGTGCACTTACCCTGTAGTACTGCTGGCACCGGCCACCGTCGGCCGTGGCGGAGGGTTACCCGGGACCACAGTGCATTTTTCC	1500
M. anisopliae TYPE ARSEF 7487	GGTGGTGCAGTTACAGCAGTGCACTTACCCTGTAGTACTGCTGGCACCGGCCACCGTCGGCCGTGGCGGAGGGTTACCCGGGACCACAGTGCATTTTTCC	1500
M. anisopliae ARSEF 7450	GGTGGTGCAGTTACAGCAGTGCACTTACCCTGTAGTACTGCTGGCACCGGCCACCGTCGGCCGTGGCGGAGGGTTACCCGGGACCACAGTGCATTTTTCC	1500
M. anisopliae ARSEF 2080	GGTGGTGCAGTTACAGCAGTGCACTTACCCTGTAGTACTGCTGGCACCGGCCACCGTCGGCCGTGGCGGAGGGTTACCCGGGACCACAGTGCATTTTTCC	1500
manihyb-IDF2	--	1600
mani-IDR2 anti	--	1600
mhyb-IDR2 anti	--	1600
M. hybridum TYPE ARSEF 549	GTTTTTTCCATTTTTTTCAAAATCGACGACCAAAAAGTTGGTAGGTCTGGGTTGCCTTGCTGGCCAATAGAGATTTGTATGGCGCTACTATAGGGTAGGCT	1600
M. hybridum ARSEF 1080	GTTTTTTCCATTTTTTTCAAAATCGACGACCAAAAAGTTGGTAGGTCTGGGTTGCCTTGCTGGCCAATAGAGATTTGTATGGCGCTACTATAGGGTAGGCT	1600
M. hybridum ARSEF 6347	GTTTTTTCCATTTTTTTCAAAATCGACGACCAAAAAGTTGGTAGGTCTGGGTTGCCTTGCTGGCCAATAGAGATTTGTATGGCGCTACTATAGGGTAGGCT	1600
M. anisopliae TYPE ARSEF 7487	GTTTTTTCCATTTTTTTCAAAATCGACGACCAAAAAGTTGGTAGGTCTGGGTTGCCTTGCTGGCCAATAGAGATTTGTAT-----------AGGGTAGGCC	1600
M. anisopliae ARSEF 7450	GTTTTTTCCATTTTTTTCAAAATCGACGACCAAAAAGTTGGTAGGTCTGGGTTGCCTTGCTGGCCAATAGAGATTTGTAT-----------AGGGTAGGCC	1600
M. anisopliae ARSEF 2080	GTTTTTTCCATTTTTTTCAAAATCGACGACCAAAAAGTTGGTAGGTCTGGGTTGCCTTGCTGGCCAATAGAGATTTGTAT-----------AGGGTAGGCC	1600

```
manihyb-IDF2                  ---------------------------------------------------------------------------------------------------- 1700
mani-IDR2 anti                -------------------------------------------------CTCCGCGCGAATGGCAGT--------------------------------- 1700
mhyb-IDR2 anti                -------------------------------------------------CTC----------GCAGTTTGGTAGGGC----------------------- 1700
M. hybridum TYPE ARSEF 549    GTGCC-GGTACTTGCTGTCGCATACAAATTCCCTAGTAACTGGAAGTACCTC----------GCAGTTTGGTAGGGCAGGTACCGGGTAGGCTG-TGCCG 1700
M. hybridum ARSEF 1080        GTGCC-GGTACTTGCTGTCGCATACAAATTCCCTAGTAACTGGAAGTACCTC----------GCAGTTTGGTAGGGCAGGTACCGGGTAGGCTG-TGCCG 1700
M. hybridum ARSEF 6347        GTGCC-GGTACTTGCTGTCGCATACAAATTCCCTAGTAACTGGAAGTACCTC----------GCAGTTTGGTAGGGCAGGTACCGGGTAGGCTG-TGCCG 1700
M. anisopliae TYPE ARSEF 7487 GGGGCAGGTACTTGCAGTCGCATACAAATTCTCTAGTAATTTGATGTACCTCCGCGCGAATGGCAGTTTGGCAGGGTAGGTACCGGGTAGGCTGGTGCAG 1700
M. anisopliae ARSEF 7450      GGGGCAGGTACTTGCAGTCGCATACAAATTCTCTAGTAATTTGATGTACCTCCGCGCGAATGGCAGTTTGGCAGGGTAGGTACCGGGTAGGCTGGTGCAG 1700
M. anisopliae ARSEF 2080      GGGGCAGGTACTTGCAGTCGCATACAAATTCTCTAGTAATTTGATGTACCTCCGCGCGAATGGCAGTTTGGCAGGGTAGGTACCGGGTAGGCTGGTGCAG 1700

manihyb-IDF2                  ------------------------------------------------------------- 1761
mani-IDR2 anti                ------------------------------------------------------------- 1761
mhyb-IDR2 anti                ------------------------------------------------------------- 1761
M. hybridum TYPE ARSEF 549    GTACTTGCTGTCGCATACAAATTCCCTAGTAACTGGAAGTACCCCCGCGCGCGGGCAACTT 1761
M. hybridum ARSEF 1080        GTACTTGCTGTCGCATACAAATTCCCTAGTAACTGGAAGTACCCCCGCGCGCGGGCAACTT 1761
M. hybridum ARSEF 6347        GTACTTGCTGTCGCATACAAATTCCCTAGTAACTGGAAGTACCCCCGCGCGCGGGCAACTT 1761
M. anisopliae TYPE ARSEF 7487 GTACTTGCAGTTGCATACAAATTCTCTAGTAACTGGAAGTACCCCCGCGCGCGGGCAACTT 1761
M. anisopliae ARSEF 7450      GTACTTGCAGTTGCATACAAATTCTCTAGTAACTGGAAGTACCCCCGCGCGCGGGCAACTT 1761
M. anisopliae ARSEF 2080      GTACTTGCAGTTGCATACAAATTCTCTAGTAACTGGAAGTACCCCCGCGCGCGGGCAACTT 1761
```

Supplementary Figure S8. Alignment of ribosomal intergenic spacer (rIGS) sequences used for the design of species-discriminating primers for *Metarhizium hybridum* and *Metarhizium anisopliae*. Diagnostic primer sequences are marked yellow.
