# Supplementary material for: Species-Discriminating Diagnostic PCR, Ribosomal Intergenic Spacer-Based Single-Marker Taxonomy and Cryptic Descriptions of the Fungal Entomopathogens Metarhizium hybridum and Metarhizium parapingshaense
Source: J Fungi (Basel). 2026 Apr 9;12(4):272. doi: 10.3390/jof12040272 (PMC13117108; doi:10.3390/jof12040272)
Supplement: Supplementary file 1 [file jof-12-00272-s001.zip › Suppl Figure S9.pdf]

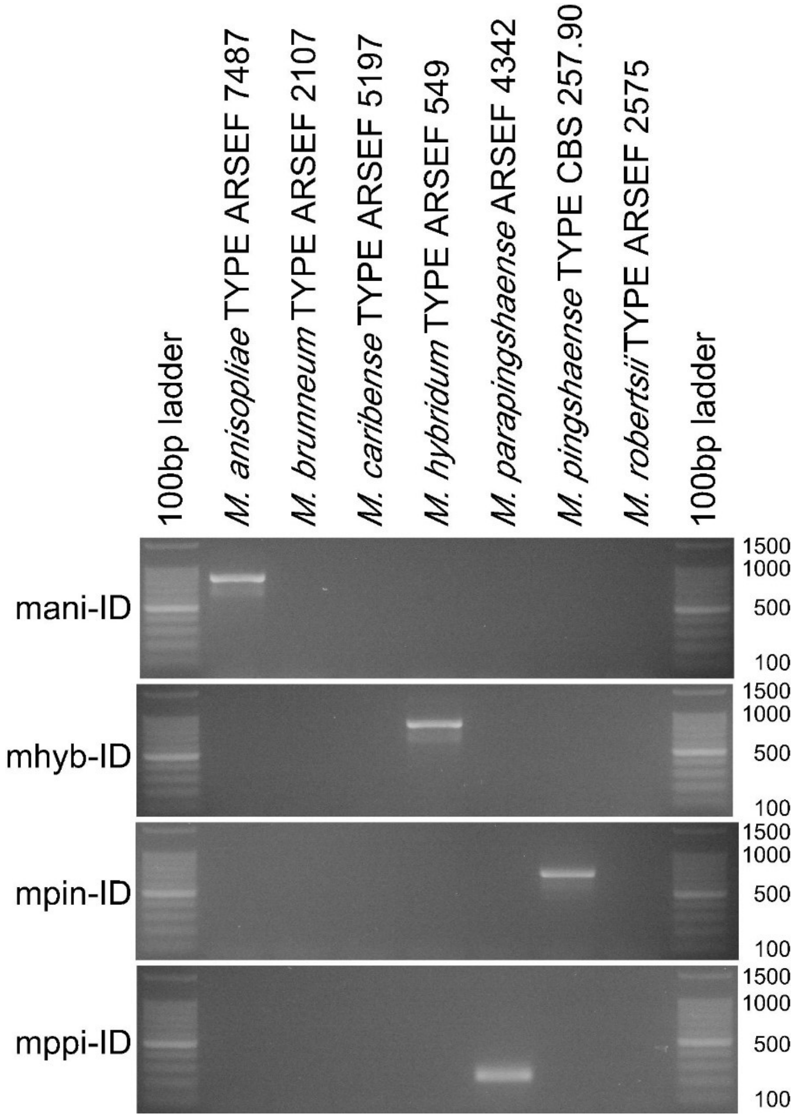

Supplementary Figure S9. Agarose gel electrophoresis of diagnostic PCRs using species-discriminating primer pairs mani-ID, mhyb-ID, mpin-ID and mppi-ID specific for *M. anisopliae, M. hybridum, M. pingshaense* and *M. parapingshaense*, respectively, as indicated at the left margin. The length (in bp) of main signals in the size standard is indicated at the right margin. Lane labels on top of the picture designate the *Metarhizium* species and strain; "TYPE" indicates nomenclatural type strains. "100bp ladder" denotes the size standard.
