# Supplementary material for: Species-Discriminating Diagnostic PCR, Ribosomal Intergenic Spacer-Based Single-Marker Taxonomy and Cryptic Descriptions of the Fungal Entomopathogens Metarhizium hybridum and Metarhizium parapingshaense
Source: J Fungi (Basel). 2026 Apr 9;12(4):272. doi: 10.3390/jof12040272 (PMC13117108; doi:10.3390/jof12040272)
Supplement: Supplementary file 1 [file jof-12-00272-s001.zip › Suppl Table S1.pdf]

Supplementary Table S1. *Metarhizium* strains and marker sequences referred to in this study. "-T" following a strain designation denotes a nomenclatural type strain.

Species Assignment	Strain Designation	GenBank accession numbers					Geographic Origin	Original Host / Isolation Substrate
		rIGS	5TEF	DUF895	EF1A	RPB2		
Metarhizium anisopliae	ARSEF 2080	PV754230					Indonesia	*Nilaparvata lugens* (Hemiptera: Delphacidae)
Metarhizium anisopliae	ARSEF 7450	MH604977					Australia	Coleoptera
Metarhizium anisopliae	ARSEF 7487 - T	MH604974	DQ463996	KC164558	DQ463996	DQ468370	Ethiopia	Orthoptera
Metarhizium anisopliae	LQUZB-13	PZ012312					Uzbekistan	soil
Metarhizium anisopliae	LQUZB-18	PZ012313					Uzbekistan	soil
Metarhizium anisopliae	LQUZB-7	PZ012311					Uzbekistan	soil
Metarhizium brunneum	ARSEF 2107 - T	MH604983	EU248855	KC164556	EU248855	EU248935	U.S.A.	Coleoptera
Metarhizium caribense	ARSEF 5197 - T	PP034621	MH712766	KX342465	PP034604	PP034642	U.S.A.	*Diaprepes abbreviata* (Coleoptera: Curculionidae)
Metarhizium guizhouense	CBS 258.90 - T	MH604996	EU248862	KU980515	EU248862	EU248942	China	Lepidoptera
Metarhizium humberi	ESALQ 1638	JACEFI010000005.1		KP028040			Brazil	soil
Metarhizium humberi	IP-46 - T	-	JQ061205		MH837574	MH837565	Brazil	soil
Metarhizium hybridum	ARSEF 1080	OR296678					U.S.A.	*Helicoverpa zea*
Metarhizium hybridum	ARSEF 549 - T	PV754229	PZ016460	PZ016461	PZ016462	PZ016463	Brazil	-
Metarhizium hybridum	ARSEF 6347	MH604976					Colombia	*Hemiptera*
Metarhizium hybridum	ARSEF 798	OR296677					Colombia	*Aeneolamia varia*
Metarhizium hybridum	CEPAVE CEP 076	OR441066					Argentina	*Kanaima fluvialis* (Hemiptera: Cercopidae)
Metarhizium hybridum	CEPAVE CEP 085	PV341579					Argentina	*Kanaima fluvialis* (Hemiptera: Cercopidae)
Metarhizium hybridum	CEPAVE CEP 120	OR441067					Argentina	*Kanaima fluvialis* (Hemiptera: Cercopidae)
Metarhizium hybridum	CEPAVE CEP 160	PV341580					Argentina	Hemiptera: Cercopidae in *Eryngium* sp.
Metarhizium hybridum	INISAV LBM-10	OR441060					Cuba	undetermined Hemipteran insect

Metarhizium hybridum	INISAV LBM-11	OR441061					Cuba	*Mocis latipes* (Lepidoptera: Erebidae)
Metarhizium hybridum	INISAV LBM-12	OR441062					Cuba	*Corcyra cephalonica* (Lepidoptera: Pyralidae)
Metarhizium hybridum	INISAV LBM-267	OR441064					Cuba	Leaf hopper (Hemiptera: Cicadellidae)
Metarhizium hybridum	INISAV LBM-5	OR441058					Cuba	undetermined Hemipteran insect
Metarhizium majus	ARSEF 1914 - T	MH604993	EU248868	KC164555	KJ398801	KJ398708	Phillipines	Coleoptera
Metarhizium parapingshaense	ARSEF 3180	PP034623					Philippines	soil
Metarhizium parapingshaense	ARSEF 4290	PP034625					Solomon Islands	Coleoptera
Metarhizium parapingshaense	ARSEF 4342	MH604980	EU248851	LC709291	EU248851	EU248931	Solomon Islands	Coleoptera
Metarhizium parapingshaense	BCC 37941 - T	SRX24959043					Thailand	insect cadaver (Diptera) from Dry Evergreen Forest
Metarhizium parapingshaense	BCC 96582	SRX24959087					Thailand	fruit orchard soil
Metarhizium pingshaense	ARSEF 2231	PP034622					India	*Zygogramma bicolorata*
Metarhizium pingshaense	ARSEF 2809	OR296682					Philippines	soil
Metarhizium pingshaense	ARSEF 3210	MH604981					India	Coleoptera
Metarhizium pingshaense	ARSEF 7929	MH604979					Australia	Isoptera
Metarhizium pingshaense	ARSEF 9975	PP034626					U.S.A.	soil
Metarhizium pingshaense	CBS 257.90 - T	MH604978	EU248850	KC164559	EU248850	OR296676	China	Coleoptera
Metarhizium robertsii	ARSEF 2575 - T	MH604971	KX342729	KX342480	KR706486	JELW01000009.1	U.S.A.	*Curculio caryae*
