# Supplementary material for: Species-Discriminating Diagnostic PCR, Ribosomal Intergenic Spacer-Based Single-Marker Taxonomy and Cryptic Descriptions of the Fungal Entomopathogens Metarhizium hybridum and Metarhizium parapingshaense
Source: J Fungi (Basel). 2026 Apr 9;12(4):272. doi: 10.3390/jof12040272 (PMC13117108; doi:10.3390/jof12040272)
Supplement: Supplementary file 1 [file jof-12-00272-s001.zip › Suppl Table S2.pdf]

Supplementary Table S2. Preparative PCR primers used and reaction-specific parameters applied in this study.

Primer Designation	Primer Sequence (5′ => 3′)	Annealing Temperature (°C)	Elongation Time (sec)	References
Migs1-F1	CCTTGTTGTTACGATCTGCTGAG	55	90	[13]
Migs850-R1	TAAASAAGCAGCCTACCCTAAAGC			
Ma-28S4f	CCTTGTTGTTACGATCTGCTGAGGG	58	300	[12]
Ma-18S4r	TAATGAGCCATTCGCAGTTTCGCTG			
