# Supplementary material for: Species-Discriminating Diagnostic PCR, Ribosomal Intergenic Spacer-Based Single-Marker Taxonomy and Cryptic Descriptions of the Fungal Entomopathogens Metarhizium hybridum and Metarhizium parapingshaense
Source: J Fungi (Basel). 2026 Apr 9;12(4):272. doi: 10.3390/jof12040272 (PMC13117108; doi:10.3390/jof12040272)
Supplement: Supplementary file 1 [file jof-12-00272-s001.zip › Suppl Table S3.pdf]

**Supplementary Table S3.** Cryptic Descriptions of *Metarhizium hybridum* and *Metarhizium parapingshaense* identified in the GenBank database. "n.a." abbreviates "data not available".

| *Revised Taxonomic Assignment* | *Previous Taxonomic Assignment* | **Strain Designation** | **rIGSID800** | **5TEF** | **DUF895** | **EF1A** | **RPB2** | **Geographic Origin** | **Original Host / Isolation Substrate** |
|---|---|---|---|---|---|---|---|---|---|
| *Metarhizium hybridum* | *Metarhizium acridum* | CG423 | AY847480 | | | | | Brazil | *Schistocerca pallens* (Orthoptera: Acrididae) |
| *Metarhizium hybridum* | *Metarhizium album* | CG515 | AY847496 | | | | | Philippines | *Nephotettix virescens* (Homoptera: Cicadellidae) |
| *Metarhizium hybridum* | *Metarhizium anisopliae* | AGF52 | | MK054159 | | | | New Zealand | n.a. |
| *Metarhizium hybridum* | *Metarhizium anisopliae* | ARSEF 1045 | | | KX342448 | | | Brazil | n.a. |
| *Metarhizium hybridum* | *Metarhizium anisopliae* | ARSEF 1080 | OR296678 | | | | OR296665 | USA | *Helicoverpa zea* |
| *Metarhizium hybridum* | *Metarhizium anisopliae* | ARSEF 6347 | MH604976 | | | | | Colombia | Hemiptera |
| *Metarhizium hybridum* | *Metarhizium anisopliae* | ARSEF 798 | OR296677 | | KX342447 | OR296642 | OR296664 | Colombia | *Aeneolamia varia* |
| *Metarhizium hybridum* | *Metarhizium anisopliae* | BV-HE-23 | | OK041470 | | | | Mexico | Coconut tree |
| *Metarhizium hybridum* | *Metarhizium anisopliae* | C2EF | | MZ712199 | | | | Brazil | n.a. |
| *Metarhizium hybridum* | *Metarhizium anisopliae* | CEP 076 | OR441066 | OR441044 | | | | Argentina | *Kanaima fluvialis* (Hemiptera: Cercopidae) |
| *Metarhizium hybridum* | *Metarhizium anisopliae* | CEP 120 | OR441067 | OR441045 | | | | Argentina | *Kanaima fluvialis* (Hemiptera: Cercopidae) |
| *Metarhizium hybridum* | *Metarhizium anisopliae* | CEPAF_ENT_25 | | OR250326 | OR250315 | | | Brazil | soil |
| *Metarhizium hybridum* | *Metarhizium anisopliae* | CEPAF_ENT_27 | | | OR250317 | | | Brazil | soil |
| *Metarhizium hybridum* | *Metarhizium anisopliae* | CEPAF_ENT_29 | | OR250330 | OR250318 | | | Brazil | soil |
| *Metarhizium hybridum* | *Metarhizium anisopliae* | CEPAF_ENT_39 | | OR250333 | | | | Brazil | soil |
| *Metarhizium hybridum* | *Metarhizium anisopliae* | CEPAF_ENT_42 | | | OR250320 | | | Brazil | soil |
| *Metarhizium hybridum* | *Metarhizium anisopliae* | CEPAF_ENT_46 | | OR250335 | OR250321 | | | Brazil | soil |
| *Metarhizium hybridum* | *Metarhizium anisopliae* | CEPAF_ENT_58 | | OR250337 | OR250323 | | | Brazil | soil |
| *Metarhizium hybridum* | *Metarhizium anisopliae* | CG143 | AY847484 | | | | | Brazil | Homoptera: Cercopidae |
| *Metarhizium hybridum* | *Metarhizium anisopliae* | CG144 | AY847490 | | | | | Brazil | *Piezodorus guildinii* (Hemiptera: Pentatomidae) |
| *Metarhizium hybridum* | *Metarhizium anisopliae* | CG148 | | MK392371 | | | | Brazil | *Deois flavopicta* (Homoptera: Cercopidae) |
| *Metarhizium hybridum* | *Metarhizium anisopliae* | CG153 | AY847503 | | | | | Brazil | *Deois incompleta* (Homoptera: Cercopidae) |
| *Metarhizium hybridum* | *Metarhizium anisopliae* | CG28 (AL) | AY847492 | | | | | Brazil | *Mahanarva posticata* (Homoptera: Cercopidae) |
| *Metarhizium hybridum* | *Metarhizium anisopliae* | CG31 | AY847499 | | | | | Brazil | *Deois flavopicta* (Homoptera: Cercopidae) |
| *Metarhizium hybridum* | *Metarhizium anisopliae* | CG33 | AY847498 | | | | | Brazil | *Deois flavopicta* (Homoptera: Cercopidae) |
| *Metarhizium hybridum* | *Metarhizium anisopliae* | CG340 | AY847500 | | | | | Brazil | *Mahanarva posticata* (Homoptera: Cercopidae) |
| *Metarhizium hybridum* | *Metarhizium anisopliae* | CG37 | AY847497 | | | | | Brazil | soil |
| *Metarhizium hybridum* | *Metarhizium anisopliae* | CG39 | AY847493 | | | | | Brazil | soil |
| *Metarhizium hybridum* | *Metarhizium anisopliae* | CG40 | AY847505 | | | | | Brazil | *Deois flavopicta* (Homoptera: Cercopidae) |
| *Metarhizium hybridum* | *Metarhizium anisopliae* | CG41 | AY847511 | | | | | Brazil | *Nezara viridula* (Hemiptera: Pentatomidae) |
| *Metarhizium hybridum* | *Metarhizium anisopliae* | CG419 | AY847501 | | | | | Brazil | soil |
| *Metarhizium hybridum* | *Metarhizium anisopliae* | CG43 | AY847502 | | | | | Brazil | *Zulia entreriana* (Homoptera: Cercopidae) |
| *Metarhizium hybridum* | *Metarhizium anisopliae* | CG46 (E9) | AY847488 | | | | | Brazil | *Deois incompleta* (Homoptera: Cercopidae) |
| *Metarhizium hybridum* | *Metarhizium anisopliae* | CG491 | AY847485 | | | | | Brazil | *Deois sp.* (Homoptera: Cercopidae) |
| *Metarhizium hybridum* | *Metarhizium anisopliae* | CG578 | AY847507 | | | | | Brazil | *Zulia entreriana* (Homoptera: Cercopidae) |
| *Metarhizium hybridum* | *Metarhizium anisopliae* | CG626 | AY847508 | | | | | Brazil | *Mahanarva posticata* (Homoptera: Cercopidae) |
| *Metarhizium hybridum* | *Metarhizium anisopliae* | CG858 | AY847504 | | | | | Brazil | *Mahanarva fimbriolata* (Homoptera: Cercopidae) |
| *Metarhizium hybridum* | *Metarhizium anisopliae* | CHE-CNRCB 235 | | KU725694 | KU725709 | KU725694 | KU725702 | Mexico | *Aeneolamia* sp. (Hemiptera: Cercopidae) |
| *Metarhizium hybridum* | *Metarhizium anisopliae* | E6 | JNNZ01 | | | AY445082 | | Brazil | *Deois flavopicta* (Homoptera: Cercopidae) |
| *Metarhizium hybridum* | *Metarhizium anisopliae* | E6S1 | AY847481 | | | | | n.a. | n.a. |
| *Metarhizium hybridum* | *Metarhizium anisopliae* | E6S2 | AY847483 | | | | | Brazil | *Deois flavopicta* (Homoptera: Cercopidae) |
| *Metarhizium hybridum* | *Metarhizium anisopliae* | EH467 | | KY616809 | KY024734 | | | Mexico | Hemiptera |

| | | | | | | | | | |
|---|---|---|---|---|---|---|---|---|---|
| *Metarhizium hybridum* | *Metarhizium anisopliae* | EH468 | | KY616810 | | | | Mexico | Hemiptera |
| *Metarhizium hybridum* | *Metarhizium anisopliae* | EH473 | | KY616797 | KY024736 | | | Mexico | Hemiptera |
| *Metarhizium hybridum* | *Metarhizium anisopliae* | EH477 | | KY616798 | KY024737 | | | Mexico | Hemiptera |
| *Metarhizium hybridum* | *Metarhizium anisopliae* | EH478 | | KY616799 | KY024738 | | | Mexico | Hemiptera |
| *Metarhizium hybridum* | *Metarhizium anisopliae* | EH479 | | KY616800 | KY024739 | | | Mexico | Hemiptera |
| *Metarhizium hybridum* | *Metarhizium anisopliae* | EH480 | | KY616801 | | | | Mexico | Hemiptera |
| *Metarhizium hybridum* | *Metarhizium anisopliae* | EH805 | | KY616803 | KY024746 | | | Mexico | Hemiptera |
| *Metarhizium hybridum* | *Metarhizium anisopliae* | EH849 | | KY616804 | KY024750 | | | Mexico | Lepidoptera |
| *Metarhizium hybridum* | *Metarhizium anisopliae* | EH863 | | KY616807 | KY024756 | | | Mexico | Coleoptera |
| *Metarhizium hybridum* | *Metarhizium anisopliae* | ESALQ_1037 | | | KP028015 | | | Brazil | Hymenoptera: Formicidae |
| *Metarhizium hybridum* | *Metarhizium anisopliae* | ESALQ_1175 | | KP027963 | | | | Brazil | soil |
| *Metarhizium hybridum* | *Metarhizium anisopliae* | ESALQ_1594 | | | KP028021 | | | Brazil | soil |
| *Metarhizium hybridum* | *Metarhizium anisopliae* | ESALQ_1604 | | | KP028022 | | | n.a. | n.a. |
| *Metarhizium hybridum* | *Metarhizium anisopliae* | ESALQ_3167 | | MH719674 | | | | Brazil | soil |
| *Metarhizium hybridum* | *Metarhizium anisopliae* | ESALQ_385 | | KP027953 | KP028013 | | | Brazil | Hemiptera: Cercopidae |
| *Metarhizium hybridum* | *Metarhizium anisopliae* | GZAAS5.1018 | | KC561893 | | | | China | n.a. |
| *Metarhizium hybridum* | *Metarhizium anisopliae* | INISAV LBM-10 | OR441060 | | | | MZ292447 | Cuba | Hemiptera sp. |
| *Metarhizium hybridum* | *Metarhizium anisopliae* | INISAV LBM-11 | OR441061 | | | | MZ292448 | Cuba | *Mocis latipes* (Lepidoptera: Erebidae) |
| *Metarhizium hybridum* | *Metarhizium anisopliae* | INISAV LBM-12 | OR441062 | | PP034593 | | MZ292449 | Cuba | *Corcyra cephalonica* (Lepidoptera: Pyralidae) |
| *Metarhizium hybridum* | *Metarhizium anisopliae* | INISAV LBM-267 | OR441064 | | | MZ292439 | MZ292451 | Cuba | leaf hopper (Hemiptera: Cicadellidae) |
| *Metarhizium hybridum* | *Metarhizium anisopliae* | INISAV LBM-5 | OR441058 | | PP034589 | MZ292434 | MZ292446 | Cuba | Hemiptera sp. |
| *Metarhizium hybridum* | *Metarhizium anisopliae* | LCM S12 | | ON753524 | | | | Brazil | soil |
| *Metarhizium hybridum* | *Metarhizium anisopliae* | LCM S17 | | ON753525 | | | | Brazil | soil |
| *Metarhizium hybridum* | *Metarhizium anisopliae* | LCMS01 | | MK410474 | | | | Brazil | soil |
| *Metarhizium hybridum* | *Metarhizium anisopliae* | LCMS02 | | MK410475 | | | | Brazil | soil |
| *Metarhizium hybridum* | *Metarhizium anisopliae* | LRC 202 | | MK391194 | | | | Canada | soil? |
| *Metarhizium hybridum* | *Metarhizium anisopliae* | M0 | | | | MG845573 | | Honduras | soil |
| *Metarhizium hybridum* | *Metarhizium anisopliae* | M16 | | | | MG845583 | | Costa Rica | soil |
| *Metarhizium hybridum* | *Metarhizium anisopliae* | M5 | AY847491 | | | MG845577 | | Brazil | *Deois flavopicta* (Homoptera: Cercopidae) |
| *Metarhizium hybridum* | *Metarhizium anisopliae* | M7 | | | | MG845579 | | Costa Rica | soil |
| *Metarhizium hybridum* | *Metarhizium anisopliae* | MNS1-NAY | | OP889331 | | | | n.a. | n.a. |
| *Metarhizium hybridum* | *Metarhizium anisopliae* | RCEF4241 | | | KP706742 | KM510184 | | Brazil | sugarcane spittlebug |
| *Metarhizium hybridum* | *Metarhizium anisopliae* | Rjd | AY847506 | | | | | n.a. | n.a. |
| *Metarhizium hybridum* | *Metarhizium anisopliae* | URPE-11 | | KX096871 | | | | Brazil | *Mahanarva posticata* (Homoptera: Cercopidae) |
| *Metarhizium hybridum* | *Metarhizium anisopliae* | V281 | AY847487 | | | | | Brazil | Homoptera: Cercopidae |
| *Metarhizium hybridum* | *Metarhizium anisopliae* | V285 | AY847486 | | | | | Philippines | Coleoptera |
| *Metarhizium hybridum* | *Metarhizium sp.* | URM 5946 | | PQ166345 | | | PQ156568 | Brazil | *Mahanarva fimbriolata* (Homoptera: Cercopidae) |
| *Metarhizium hybridum* | *Metarhizium sp.* | URM 5947 | | PQ166346 | | | | Brazil | *Mahanarva fimbriolata* (Homoptera: Cercopidae) |
| *Metarhizium hybridum* | *Metarhizium sp.* | URM 5949 | | | | | PQ156570 | Brazil | *Mahanarva fimbriolata* (Homoptera: Cercopidae) |
| *Metarhizium hybridum* | *Metarhizium sp.* | URM 5951 | | PQ166347 | | | | Brazil | *Mahanarva fimbriolata* (Homoptera: Cercopidae) |
| *Metarhizium hybridum* | *Metarhizium sp.* | URM 5952 | | PQ166348 | | | | Brazil | *Mahanarva fimbriolata* (Homoptera: Cercopidae) |
| *Metarhizium hybridum* | *Metarhizium sp.* | URM 6033 | | PQ166349 | | | | Brazil | *Mahanarva fimbriolata* (Homoptera: Cercopidae) |
| *Metarhizium hybridum* | *Metarhizium sp.* | URM 6034 | | PQ166350 | | | PQ156571 | Brazil | *Mahanarva fimbriolata* (Homoptera: Cercopidae) |
| *Metarhizium hybridum* | *Metarhizium sp.* | URM 6035 | | | | | PQ156567 | Brazil | *Mahanarva fimbriolata* (Homoptera: Cercopidae) |
| *Metarhizium hybridum* | *Metarhizium sp.* | URM 6096 | | PQ166352 | | | | Brazil | *Mahanarva fimbriolata* (Homoptera: Cercopidae) |
| *Metarhizium hybridum* | *Metarhizium sp.* | URM 6097 | | PQ166353 | | | | Brazil | *Mahanarva fimbriolata* (Homoptera: Cercopidae) |
| *Metarhizium hybridum* | *Metarhizium sp.* | URM 6098 | | PQ166354 | | | | Brazil | *Mahanarva fimbriolata* (Homoptera: Cercopidae) |
| *Metarhizium hybridum* | *Metarhizium sp.* | URM 6099 | | PQ166355 | | | | Brazil | *Mahanarva fimbriolata* (Homoptera: Cercopidae) |
| *Metarhizium hybridum* | *Metarhizium sp.* | URM 6100 | | PQ166356 | | | | Brazil | *Mahanarva fimbriolata* (Homoptera: Cercopidae) |
| *Metarhizium hybridum* | *Metarhizium sp.* | URM 6101 | | PQ166357 | | | | Brazil | *Mahanarva fimbriolata* (Homoptera: Cercopidae) |
| *Metarhizium hybridum* | *Metarhizium sp.* | URM 6103 | | PQ166358 | | | | Brazil | *Mahanarva fimbriolata* (Homoptera: Cercopidae) |
| *Metarhizium hybridum* | *Metarhizium sp.* | URM 6104 | | PQ166359 | | | | Brazil | *Mahanarva fimbriolata* (Homoptera: Cercopidae) |

| | | | | | | | | | |
|---|---|---|---|---|---|---|---|---|---|
| *Metarhizium hybridum* | *Metarhizium sp.* | URM 6105 | | PQ166360 | | | PQ156565 | Brazil | *Mahanarva fimbriolata* (Homoptera: Cercopidae) |
| *Metarhizium hybridum* | *Metarhizium sp.* | URM 6107 | | PQ166361 | | | | Brazil | *Mahanarva fimbriolata* (Homoptera: Cercopidae) |
| *Metarhizium hybridum* | *Metarhizium sp.* | URM 6108 | | PQ166362 | | | | Brazil | *Mahanarva fimbriolata* (Homoptera: Cercopidae) |
| *Metarhizium hybridum* | *Metarhizium sp.* | URM 6109 | | PQ166363 | | | | Brazil | *Mahanarva fimbriolata* (Homoptera: Cercopidae) |
| *Metarhizium hybridum* | *Metarhizium sp.* | URM 6111 | | PQ166364 | | | | Brazil | *Mahanarva fimbriolata* (Homoptera: Cercopidae) |
| *Metarhizium hybridum* | *Metarhizium sp.* | URM 6113 | | PQ166365 | | | | Brazil | soil |
| *Metarhizium hybridum* | *Metarhizium sp.* | URM 6114 | | PQ166366 | | | | Brazil | *Mahanarva fimbriolata* (Homoptera: Cercopidae) |
| *Metarhizium hybridum* | *Metarhizium sp.* | URM 6115 | | PQ166367 | | | | Brazil | *Mahanarva fimbriolata* (Homoptera: Cercopidae) |
| *Metarhizium hybridum* | *Metarhizium sp.* | URM 6129 | | PQ166369 | | | | Brazil | *Mahanarva fimbriolata* (Homoptera: Cercopidae) |
| *Metarhizium hybridum* | *Metarhizium sp.* | URM 6130 | | PQ166370 | | | | Brazil | *Mahanarva fimbriolata* (Homoptera: Cercopidae) |
| *Metarhizium hybridum* | *Metarhizium sp.* | URM 6131 | | PQ166371 | | | | Brazil | *Mahanarva fimbriolata* (Homoptera: Cercopidae) |
| *Metarhizium hybridum* | *Metarhizium sp.* | URM 6132 | | PQ166372 | | | | Brazil | *Mahanarva fimbriolata* (Homoptera: Cercopidae) |
| *Metarhizium hybridum* | *Metarhizium sp.* | URM 6133 | | PQ166373 | | | | Brazil | *Mahanarva fimbriolata* (Homoptera: Cercopidae) |
| *Metarhizium hybridum* | *Metarhizium sp.* | URM 6210 | | PQ166374 | | | | Brazil | *Mahanarva fimbriolata* (Homoptera: Cercopidae) |
| *Metarhizium hybridum* | *Metarhizium sp.* | URM 8139 | | MZ394804 | | | | Brazil | n.a. |
| *Metarhizium hybridum* | *Metarhizium sp.* | URM 8140 | | MZ394805 | | | | Brazil | n.a. |
| *Metarhizium hybridum* | *Metarhizium sp.* | URM 8141 | | MZ394806 | | | | Brazil | n.a. |
| *Metarhizium hybridum* | *Metarhizium sp.* | URM 8142 | | MZ394807 | | | | Brazil | n.a. |
| *Metarhizium hybridum* | *Metarhizium sp.* | URM 8143 | | MZ394808 | | | | Brazil | n.a. |
| *Metarhizium hybridum* | *Metarhizium sp.* | URM 8144 | | MZ394809 | | | | Brazil | n.a. |
| *Metarhizium hybridum* | *Metarhizium sp.* | V269 | AY847482 | | | | | n.a. | n.a. |
| *Metarhizium parapingshaense* | *Metarhizium anisopliae* | GZAAS5.1004 | | KC561895 | | | | China | n.a. |
| *Metarhizium parapingshaense* | *Metarhizium anisopliae* | RCEF6252 | | | KP706764 | | | China | soil |
| *Metarhizium parapingshaense* | *Metarhizium anisopliae* | RCEF6287 | | | KP706755 | | | China | soil |
| *Metarhizium parapingshaense* | *Metarhizium pingshaense* | ARSEF 3180 | PP034623 | | KX342455 | PP034610 | | Philippines | soil |
| *Metarhizium parapingshaense* | *Metarhizium pingshaense* | ARSEF 4290 | PP034625 | | KX342456 | | | Solomon Islands | Coleoptera |
| *Metarhizium parapingshaense* | *Metarhizium pingshaense* | F2049 | | LC188961 | | | | Japan | Cydnidae |
| *Metarhizium parapingshaense* | *Metarhizium pingshaense* | GKVK 02_40 | | KM091895 | KJ879515 | | | India | soil under cardomom |
| *Metarhizium parapingshaense* | *Metarhizium pingshaense* | GKVK 02_41 | | | KJ879516 | | | India | soil under cardomom |
| *Metarhizium parapingshaense* | *Metarhizium pingshaense* | GKVK 02_42 | | | KJ879517 | | | India | soil under cardomom |
| *Metarhizium parapingshaense* | *Metarhizium pingshaense* | GKVK 02_46 | | | KJ879520 | | | India | soil under cardomom |
| *Metarhizium parapingshaense* | *Metarhizium pingshaense* | GKVK 02_49 | | | KJ879521 | | | India | soil under cardomom |
| *Metarhizium parapingshaense* | *Metarhizium pingshaense* | JZF-014 | | | MG384146 | | | China | soil |
| *Metarhizium parapingshaense* | *Metarhizium pingshaense* | Kgs17-2 | | AB550747 | LC128485 | | | Japan | soil |
| *Metarhizium parapingshaense* | *Metarhizium pingshaense* | Myz3-2 | | | LC128489 | | | Japan | soil |
| *Metarhizium parapingshaense* | *Metarhizium pingshaense* | NCHU-64 | | | | MW344957 | | China | n.a. |
| *Metarhizium parapingshaense* | *Metarhizium pingshaense* | Okn1-3 | | | LC128493 | | | Japan | soil |
| *Metarhizium parapingshaense* | *Metarhizium pingshaense* | Okn8-1 | | AB807788 | | | | Japan | soil |
| *Metarhizium parapingshaense* | *Metarhizium pingshaense* | RCEF0988 | | | | KM510185 | | China | n.a. |
| *Metarhizium parapingshaense* | *Metarhizium pingshaense* | SFC20250301-M043 | | | | PX071852 | | South Korea | Seafoam |
| *Metarhizium parapingshaense* | *Metarhizium pingshaense* | SH-003 | | | MG384136 | | | China | soil |
| *Metarhizium parapingshaense* | *Metarhizium pingshaense* | SH-048 | | | MG384179 | | | China | soil |
| *Metarhizium parapingshaense* | *Metarhizium pingshaense* | SH-049 | | | MG384180 | | | China | soil |
| *Metarhizium parapingshaense* | *Metarhizium pingshaense* | SH-054 | | | MG384185 | | | China | soil |
| *Metarhizium parapingshaense* | *Metarhizium pingshaense* | SH-057 | | | MG384188 | | | China | soil |
| *Metarhizium parapingshaense* | *Metarhizium pingshaense* | SH-141 | | | MG384273 | | | China | soil |
| *Metarhizium parapingshaense* | *Metarhizium pingshaense* | Yks10-1 | | AB807474 | LC128501 | | | Japan | soil |
| *Metarhizium parapingshaense* | *Metarhizium pingshaense* | YXZ-018 | | | MG384150 | | | China | soil |
| *Metarhizium parapingshaense* | *Metarhizium pingshaense* | YXZ-020 | | | MG384152 | | | China | soil |
| *Metarhizium parapingshaense* | *Metarhizium pingshaense* | YYC-031 | | | MG384163 | | | China | soil |
| *Metarhizium parapingshaense* | *Metarhizium sp.* | YFCC 823 | | | | OR115183 | | China | n.a. |
